# Preclinical to clinical translation for intervertebral disc repair: Effects of species‐specific scale, metabolism, and matrix synthesis rates on cell‐based regeneration

**DOI:** 10.1002/jsp2.1279

**Published:** 2023-09-07

**Authors:** Emily E. McDonnell, Niamh Wilson, Marcos N. Barcellona, Tara Ní Néill, Jessica Bagnall, Pieter A. J. Brama, Gráinne M. Cunniffe, Stacey L. Darwish, Joseph S. Butler, Conor T. Buckley

**Affiliations:** ^1^ Trinity Centre for Biomedical Engineering, Trinity Biomedical Sciences Institute, Trinity College Dublin The University of Dublin Dublin Ireland; ^2^ Discipline of Mechanical, Manufacturing and Biomedical Engineering, School of Engineering, Trinity College Dublin The University of Dublin Dublin Ireland; ^3^ School of Veterinary Medicine University College Dublin Dublin Ireland; ^4^ National Spinal Injuries Unit Mater Misericordiae University Hospital Dublin Ireland; ^5^ School of Medicine University College Dublin Dublin Ireland; ^6^ National Orthopaedic Hospital Dublin Ireland; ^7^ St Vincent's University Hospital Dublin Ireland; ^8^ Advanced Materials and Bioengineering Research (AMBER) Centre, Royal College of Surgeons in Ireland & Trinity College Dublin The University of Dublin Dublin Ireland; ^9^ Tissue Engineering Research Group, Department of Anatomy and Regenerative Medicine Royal College of Surgeons in Ireland Dublin Ireland

**Keywords:** animal models, cell therapies, in silico, metabolism, regeneration

## Abstract

**Background:**

A significant hurdle for potential cell‐based therapies is the subsequent survival and regenerative capacity of implanted cells. While many exciting developments have demonstrated promise preclinically, cell‐based therapies for intervertebral disc (IVD) degeneration fail to translate equivalent clinical efficacy.

**Aims:**

This work aims to ascertain the clinical relevance of both a small and large animal model by experimentally investigating and comparing these animal models to human from the perspective of anatomical scale and their cellular metabolic and regenerative potential.

**Materials and Methods:**

First, this work experimentally investigated species‐specific geometrical scale, native cell density, nutrient metabolism, and matrix synthesis rates for rat, goat, and human disc cells in a 3D microspheroid configuration. Second, these parameters were employed in silico to elucidate species‐specific nutrient microenvironments and predict differences in temporal regeneration between animal models.

**Results:**

This work presents in silico models which correlate favorably to preclinical literature in terms of the capabilities of animal regeneration and predict that compromised nutrition is not a significant challenge in small animal discs. On the contrary, it highlights a very fine clinical balance between an adequate cell dose for sufficient repair, through de novo matrix deposition, without exacerbating the human microenvironmental niche.

**Discussion:**

Overall, this work aims to provide a path towards understanding the effect of cell injection number on the nutrient microenvironment and the “time to regeneration” between preclinical animal models and the large human IVD. While these findings help to explain failed translation of promising preclinical data and the limited results emerging from clinical trials at present, they also enable the research field and clinicians to manage expectations on cell‐based regeneration.

**Conclusion:**

Ultimately, this work provides a platform to inform the design of clinical trials, and as computing power and software capabilities increase in the future, it is conceivable that generation of patient‐specific models could be used for patient assessment, as well as pre‐ and intraoperative planning.

## Graphical abstract

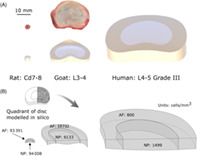

## 1 INTRODUCTION

The ultimate challenge for regenerating the intervertebral disc (IVD) has been focused on developing an easily injectable cell‐based strategy to replenish the diseased tissue with viable and functional cells. As degeneration typically manifests within the center of the IVD, these therapies are being explored as early strategies, focusing on stimulating intrinsic repair of nucleus pulposus (NP) tissue.^1^, ^2^ It is believed that these implanted healthy autologous or allogenic cells would augment extracellular matrix (ECM) anabolism, recreating the biochemical composition of the NP and subsequently restore tissue integrity and function. After showing promise in vitro, these potential new therapies must demonstrate efficacy and those benefits of the treatment outweigh its risks under preclinical assessment before progressing to clinical evaluation. Preclinical animal studies are not only crucial for understanding the progression of degeneration, how risk factors initiate, promote, or otherwise regulate degenerative changes, but also how potential therapeutics alleviate, ameliorate, or inhibit further degeneration. Moreover, animal work has been leveraged to uncover challenges likely to be encountered in a clinical setting, such as injecting cells with or without hydrogel carriers, retaining the cells in situ, and maintaining their cell viability and functionality.^3^, ^4^ Nonetheless, animal studies are expensive, labor intensive and important ethical considerations limit their widespread use. In recent years, there has been a significant drive to reduce experiments being performed on living animals through the guiding principles of Replacement, Reduction, and Refinement (the “Three Rs”). This is a systematic framework for performing more humane animal research and is a highly relevant topic across the biomedical field. Furthermore, even after demonstrating safety and efficacy in animals, many cell‐based therapies for IVD degeneration still do not appear to work to the same extent in humans.^5^ Therefore, there is a pressing need to ascertain the clinical relevance of different animal models, not only for important scientific and ethical merit but to further accelerate the prospects of more successful clinical translation.

Profound differences between animal species include cell population, both in terms of cell type and cell density, and scale of the disc geometry and structure.^6^ Large animal models have been accepted as good models for studying disc structure, geometry, biochemistry, and biomechanics.^7^, ^8^, ^9^ Larger animals have a disc structure analogous to humans and tend to undergo degeneration slowly. Examples include dogs, pigs, goats, sheep, and nonhuman primates.^10^, ^11^, ^12^, ^13^ Alternatively, small animal models such as mice, rats, and rabbits undergo degenerative changes rapidly and their popularity likely reflects their more cost‐effective and higher throughput nature.^9^, ^13^, ^14^ While large animal studies are not commonly conducted, it has been suggested that a large animal, without persisting notochordal cells (i.e., goat or sheep), is an important aspect before proceeding to human clinical trials.^3^ Vacuolated notochordal cells disappear rapidly after birth in humans and this may have implications for regenerative potential compared with species, which retain their notochordal cells into adulthood (i.e., rabbit, rat, and pig).^3^, ^6^

The human IVD consists of a sparse population of cells and an abundant ECM, which varies in matrix composition radially through the annulus fibrosus (AF), mostly type I collagen, and into the central NP, predominantly type II collagen and glycosaminoglycans (GAG). While GAG and collagen content have been comprehensively compared across the NP and AF of several animal species,^15^, ^16^ regional variation in cell density between animals has not been quantified to the same extent. Early work has reported variation in disc cell density between some animal species,^17^, ^18^ with an inverse relationship between disc cell density across the NP and disc height.^19^ However, no study has directly investigated cell density in both the NP and AF regions of both a small and large animal, as well as comparing them with respect to the sparsely populated human IVD.

Due to low oxygen conditions, disc cells are believed to primarily obtain their energy through glycolysis.^17^, ^20^ In our recent work, we highlight a number of studies which have directly measured metabolism in terms of nutrient consumption rates and/or lactate production rates (LPR) rates of NP cells.^21^ However, these investigations have typically been limited to bovine, porcine, and human cells and few studies have made a direct comparison between NP and AF cells.^22^, ^23^, ^24^, ^25^, ^26^, ^27^, ^28^, ^29^, ^30^, ^31^ While it is less experimentally challenging to measure metabolic rates in 2D culture, monolayer expansion has been shown to shift chondrocytes from a glycolytic to an oxidative energy metabolism within 7 days in vitro.^32^ Some studies have encapsulated cells in hydrogels to maintain phenotype stability during culture.^22^, ^25^, ^27^, ^31^ However, challenges remain in limiting bead or construct size, in order to mitigate gradients forming, while still maintaining sufficient cell numbers to establish detectable changes in the metabolite levels within a specified timeframe. Changes in glucose or lactate have been simply measured by sampling culture media using diabetic glucose meters or biochemical assays.^25^, ^27^, ^28^, ^29^, ^33^ Meanwhile, custom‐made or commercial metabolic chambers have been used to measure a reduction in oxygen or multiple metabolites simultaneously.^22^, ^24^, ^26^ These chambers have been reported to range from 175 μL to 4 mL in volume. Alternative more advanced Seahorse Flux Analyzer methods with greater sensitivity have been employed for rat, rabbit, and human disc cells.^34^, ^35^, ^36^, ^37^ Typically, these studies are used to investigate the effect of a substrate or inhibition of a pathway and present results in terms of oxygen consumption rate (OCR) and extracellular acidification rate (ECAR) normalized to protein content. Unfortunately, this does not facilitate comparison with rates measured using the aforementioned methods.

Since GAGs play an important role in both formation and function of the disc, they are considered fundamental to functional repair through regenerative strategies. As a result, cell‐based therapies need to be characterized by their matrix synthesizing abilities and in terms of preclinical to clinical translation, variation in the anabolic metabolism of different species needs to be considered. For example, a study by Miyazaki et al.^18^ demonstrated marked differences in proteoglycan production of notochordal (rat and rabbit) and non‐notochordal cells (bovine) in an early attempt to delineate the most suitable animal model for the study of biological repair. In order to further ascertain the clinical relevance of both a small and large animal model, this work focuses on comparing rat caudal and goat lumbar studies to human clinical trial parameters (e.g., cell injection number and study duration). First, this work will experimentally investigate and compare these animal models to human from the perspective of anatomical scale and their cellular metabolic (NP and AF cells) and regenerative potential (NP cells). Within this work, regeneration refers solely to the biosynthesis of de novo GAG matrix, a critical component to initiate restoration of the whole disc structure. Second, in silico modeling will be employed to predict the subsequent regeneration timeline of animal models compared with clinical trials. These models are governed by the aforementioned experimental parameters and can further predict the ensuing nutrient microenvironment as a result of injecting varying cell numbers into different species. Overall, this work aims to provide a path towards understanding “time to regeneration” within preclinical animal models and to elucidate the stunted success of cell‐based clinical trials.

## 2 MATERIALS AND METHODS

### 2.1 Preclinical literature and registered clinical trials of cell‐based disc regeneration

A literature search for animal models was performed in PubMed, Scopus, and Embase databases using rat search terms “intervertebral” AND “disc” AND “degeneration” AND “rat” AND (“needle puncture” OR “stab”) and goat search terms “intervertebral” AND “disc” AND “degeneration” AND “goat.” Both preclinical literature searches were refined according to Figure S1. A clinical search was performed using the clinicaltrials.gov database, a resource provided by the US National Library of Medicine. The search consisted of “IVD degeneration” as a condition or disease, “cell” as a key search term and study type was limited to “interventional studies,” which provided 50 search results. Following close inspection of each study (excluding spinal fusion interventions, cervical spine studies, and biomechanical/traction studies) the results were refined to 26 cell‐based interventions for lumbar disc degeneration.

**FIGURE 1:**
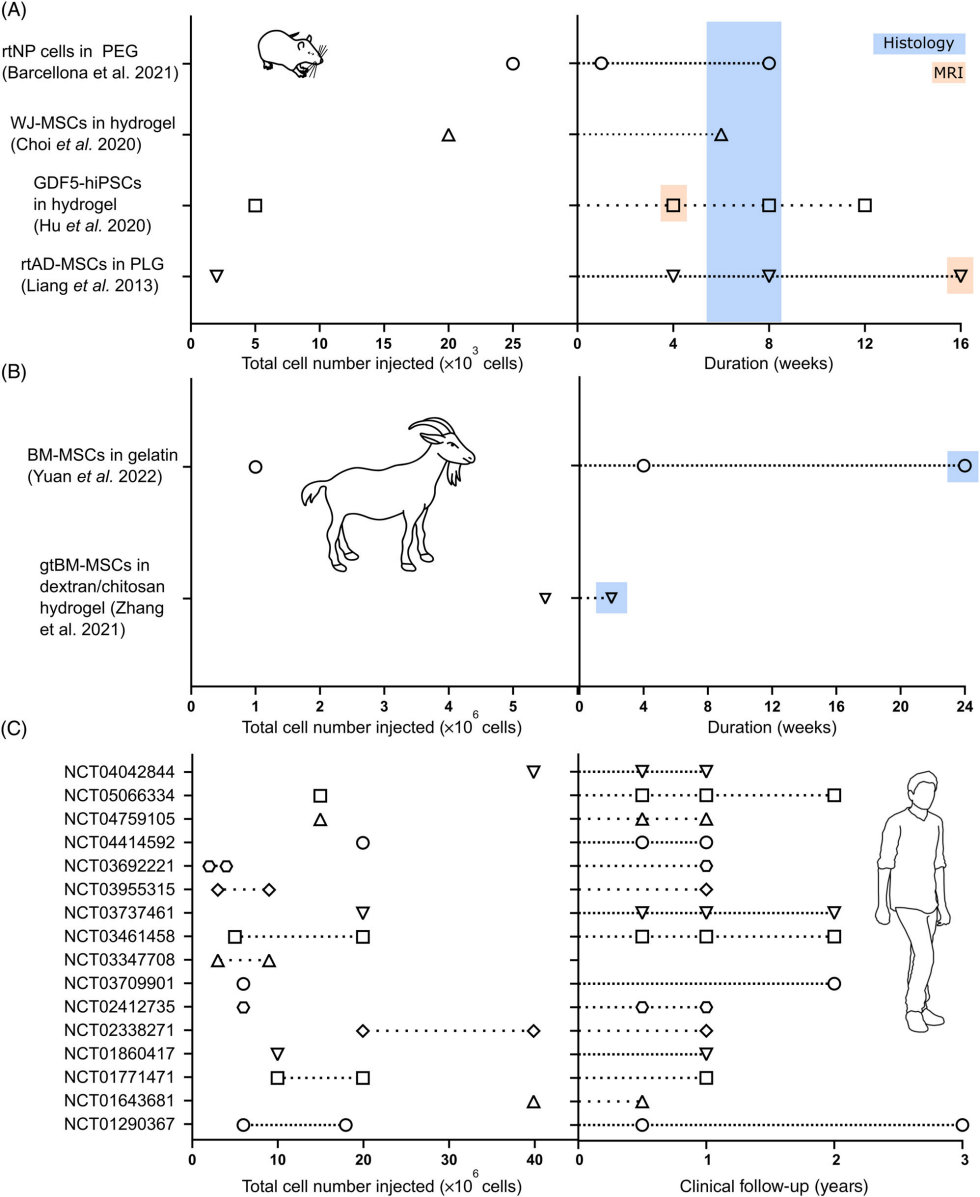
Experimental parameters gathered from key preclinical animal models in the literature and registered clinical trials for cell‐based disc regeneration. (A) Total number of cells injected and study duration for four published rat studies, highlighting the timeframe when differences were reported in histological evaluation and MRI assessment. (B) Total number of cells injected and study duration for two published goat studies, again highlighting the timeframe when differences were reported in histological evaluation. (C) Total number of cells injected across 16 registered clinical trials, with several trials using a lower and higher cell dose as indicated with dashed lines. Clinical follow‐up time points refer to functional assessment only using MRI. Abbreviations: BM‐MSCs, bone marrow‐derived mesenchymal stem cells; GDF‐5, growth differentiation factor‐5; gtBM‐MSCs, goat BM‐MSCs; hiPSCs, human induced pluripotent stem cells; PEG, polyethylene glycol; PLG, poly(lactic‐co‐glycolic acid); rtAD‐MSCs, rat adipose‐derived mesenchymal stem cells; rtNP, rat nucleus pulposus cells; WJ‐MSCs, Wharton's Jelly‐derived mesenchymal stromal cells.

From the rat models which investigated cell injection, four key studies were identified as relatively comparable in their assessment of the efficacy for a cell source delivered in a hydrogel carrier.^38^, ^39^, ^40^, ^41^ Criteria which deemed them appropriate included induced degeneration prior to treatment (at a later time point), provided details on total number of cells injected in an appropriate injection volume (<10 μL). Additionally, these studies provided clear timepoint results where improvement or significant difference to an injured control was assessed via histology and/or magnetic resonance imaging (MRI). Through a similar review of goat studies, only two key articles were identified. Compiled parameters on injected cell number and study duration (from treatment) are presented in Figure 1A,B for rat and goat, respectively. For comparison, Figure 1C presents 16 of the registered clinical trials which provided details to the same extent, for example, total cell number of a distinct cell source (i.e., exclusion of bone marrow concentrates or platelet‐rich plasma) and clinical follow‐up time points which involve MRI for functional regeneration assessment.

### 2.2 Establishing species‐specific in silico models using experimentally determined geometries and cell densities

**FIGURE 2:**
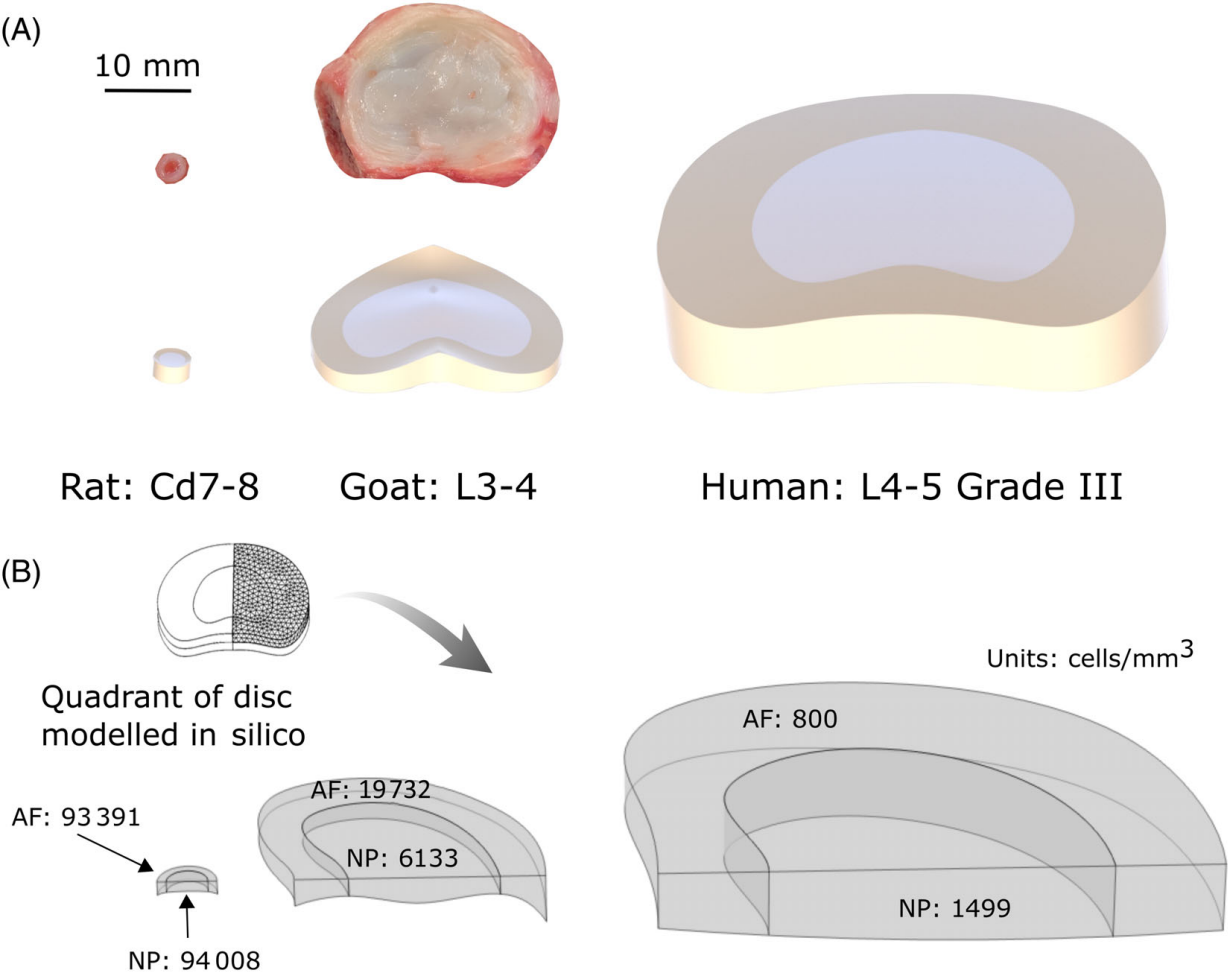
Rat caudal (Cd7‐8), goat lumbar (L3‐4) and human lumbar (L4‐5 at Grade III) discs presented in scale to one another. (A) Transverse image of a freshly isolated rat and goat disc together with 3D renderings created using measured dimensions. (B) Due to symmetry, only a quadrant of rat, goat and human discs were modeled in silico. Distinct nucleus pulposus (NP) and annulus fibrosus (AF) domains are highlighted together with their modeled native cell densities. Rat and goat cell densities were determined experimentally within this work, while human data were obtained from the literature and is specific to Grade III degeneration.^48^

To accurately create a 3D geometry for in silico models of rat caudal and goat lumbar discs, a geometrical analysis of all caudal and lumbar levels was first performed. Additionally, a literature search was carried out to identify commonly used anatomical level within animal studies. For goat, the lumbar section was excised using the last rib for anatomical reference and the L1‐2 to L5‐6 discs were isolated, dissected, photographed, and macroscopic image analysis was performed using ImageJ in the sagittal and transverse reference plane (*N* = 3). A schematic for reference can be found in Figure S2. For rat, disc levels were identified by digital palpation following removal of the skin, with the last set of palpable processes reportedly found on the fifth caudal vertebra (i.e., level Cd5) and then counting towards the sacrum to locate Cd1.^42^, ^43^ A section containing Cd1‐Cd10 was prepped for histology by fixing for 48 h in 10% formalin under gentle rotation at room temperature. Tails were then placed into a decalcifying solution until the vertebral bodies were soft (3–5 days). Sections were stained with hematoxylin and eosin (H&E) and a dichrome stain consisting of alcian blue (AB) and picrosirius red (PSR) (all Sigma–Aldrich, Ireland). The interface between NP and AF was confirmed by the transition in ECM components between the two domains, that is, changing from higher GAG staining (AB) within the NP to higher collagen (PSR) within the AF. Microscopic image analysis was then performed using ImageJ as depicted in Figure S2 (*N* = 6). An idealized 3D geometry was created using SOLIDWORKS® for rat Cd7‐8 and goat L3‐4, based on the measured dimensions and how frequently these levels are used in preclinical studies. An idealized 3D geometry for a Grade III human lumbar disc (L4‐5) was created using dimensions determined through image segmentation from MRI, graded by an expert using the Pfirrmann grading system. Full rat, goat, and human IVDs with a separate NP and AF domain are presented in Figure 2A. However, for simplification only a quadrant of the disc is modeled in silico using COMSOL Multiphysics 6.0 (COMSOL Inc., Burlington, USA), Figure 2B. Species‐specific metabolically active cell density for NP and AF was determined using methylthiazolyldiphenyl‐tetrazolium bromide (MTT) and a DAPI (4′,6‐diamidino‐2‐phenylindole) counterstain as published previously for bovine tissue.^44^

### 2.3 Cell isolation and monolayer expansion

Disc tissue was harvested from caudal spines of 6‐month‐old Wistar rats (male and female) and lumbar spines of skeletally mature (4–5 years) Saanen goats (female) within 1 and 5–6 h of sacrifice, respectively. Rat/goat tissue was obtained from discarded tissue of animals undergoing procedures approved by the Health Products and Regulatory Authority (HPRA) in the Comparative Medicine Unit (CMU), Trinity College Dublin or the School of Veterinary Medicine, University College Dublin. Under sterile conditions, NP tissue was removed first and gathered separately to the AF which was diced into smaller pieces on a sterile petri dish. Tissue from 3 to 6 rat tails was pooled into each biological repeat or “prep” to ensure sufficient cell numbers from the isolation. Tissue was enzymatically digested using a combined pronase (Millipore, Sigma) and collagenase type II (Gibco, Thermo Fisher Scientific) serum‐free digestion media. Rat tissue (NP and/or AF) was digested in 5 mL of digestion media per tail (10–12 discs/tail) and goat tissue (NP and/or AF) was digested in 10 mL per disc. Pronase was used at an activity of 70 U/mL and 100 U/mL of digestion media for rat and goat, respectively. Collagenase type II was used at an activity of 400 and 300 U/mL of digestion media for rat and goat, respectively. Rat tissue was incubated for a period of 5–6 h at 37°C under constant rotation (10 r.p.m.). Rat NP digest was triturated with an 18‐G needle as the tissue started to break up (after 1–2 h). Rat AF and both goat tissues underwent additional physical agitation every 2 h using a gentleMACS Tissue Dissociator (Miltenyi Biotech Gentlemacs Tissue Dissociator).

Human disc tissue was collected through informed consent of patients undergoing discectomy procedures and approved by the Mater Misericordiae University Hospital IRB (Ref 1/378/2229) and Trinity College Dublin (TCDFSTEMSREC/15032021/Buckley). Under sterile conditions, tissue was weighed and washed with phosphate buffered saline (PBS; 2% Pen‐Strep) by repeated centrifugation (650 × g for 5 min) and aspiration. To confirm absence of bacterial growth, tissue was placed in a 70 mL container with serum‐free low glucose Dulbecco's Modified Eagle Medium (LG‐DMEM) supplemented with 2% Pen‐Strep and 100 μg/mL kanamycin sulfate (Gibco, Thermo Fisher Scientific) and cultured overnight (37°C in a humidified atmosphere with 5% CO_2_ and 10% O_2_). Under sterile conditions, tissue was separated into NP and AF and then diced into smaller pieces on separate sterile petri dishes. Like the animal cell isolations, human NP and AF were enzymatically digested for 3–4 h using 10 mL of digestion media per g of tissue, with an enzyme activity of 100 and 300 U/mL for pronase and collagenase, respectively. All digests were stopped when there was a good proportion of single cells, rather than cell clusters, visible under a hemocytometer. The remaining suspension was passed through a cell strainer (70 μm), filtrate was centrifuged and rinsed with PBS before determining cell yield and seeding at a cell density of 5 × 10^3^ cells/cm^2^. Disc cells were expanded in LG‐DMEM supplemented with 10% fetal bovine serum (FBS) and 2% Pen‐Strep (all Gibco, Thermo Fisher Scientific). Cells were expanded no more than passage 2 (P2) with medium exchanges performed every 3 days (37°C in a humidified atmosphere with 5% CO_2_ and 10% O_2_).

### 2.4 Establishing a spheroid culture system for rat, goat, and human disc cells

Positive mold stamps were designed using SOLIDWORKS® and the individual microwell design was based on recent microwell literature.^45^, ^46^ Each microwell had a diameter of 1 mm and a total depth of 1.5 mm, comprising a cylindrical section (1 mm) and a domed end (0.5 mm), Figure S3. An array of microwells was patterned across the circular base of a stamp designed to fit into a 24‐well plate (69 microwells per well). The positive mold stamp was fabricated using a Form 2 stereolithography printer and V2 high temperature resin (Formlabs). High temperature resin was chosen to allow for autoclave sterilization. To fabricate negative hydrogel microwells under sterile conditions, 1 mL of molten agarose (2% [w/v] at ~80°C, Sigma‐Aldrich) was pipetted into wells of a 24‐well plate. After the agarose had cooled and solidified, the stamp was carefully removed, leaving behind a hydrogel microwell array in the well plate as shown in Figure S3B. Expanded NP and AF cells were seeded into the microwell array by pipetting an appropriate density into each 24‐well. After seeding, plates were incubated to allow cells to accumulate into the individual microwells (~20 min) before centrifuging at 850 × g for 5 min to condense cells at the bottom of each well (Figure S3C). Spheroids were then cultivated in LG‐DMEM supplemented with 10% FBS and 2% Pen‐Strep (all Gibco) with a daily media exchange for 5–7 days (37°C in a humidified atmosphere with 5% CO_2_ and 10% O_2_). External incubator oxygen, media glucose concentration, and regularity of the media exchange were informed through predictive in silico models as published in our previous work.^21^, ^47^ As shown in Figure S4, a daily media exchange of LG‐DMEM was necessary to prevent glucose concentrations from reducing by >50% between more standardized biweekly media exchanges. Based on in vivo concentrations consolidated previously,^48^ average local concentrations of approximately pH 7 and 5% O_2,_ across the species‐specific spheroids, were considered to be more physiologically representative and established through the aforementioned external boundary conditions. Cell spheroid viability was established using a live/dead assay kit (Invitrogen, Bioscience). Media was aspirated and hydrogel microwells were gently rinsed with PBS before incubation for 1 h in a phenol‐free DMEM (Sigma) solution containing 2 μM calcein AM and 4 μM of ethidium homodimer‐1 (EthD‐1). Following incubation, hydrogel microwells were rinsed and spheroids were dislodged from their individual microwells. Samples were imaged on a Leica SP8 scanning confocal microscope (485 and 530 nm excitation and 530 and 645 nm emission for calcein and EthD‐1, respectively). All images are presented as maximum projection z‐stack reconstructions qualitatively analyzing cell viability.

### 2.5 Experimentally determining species‐specific metabolic rates and GAG synthesis rates

The Seahorse XFe96 analyzer, together with Spheroid FluxPak (Agilent Technologies), simultaneously measures in real time the reduction in oxygen level, a measure of OCR and pH level, a measure of ECAR in the medium directly surrounding a single cell spheroid. Seahorse cartridge plates were hydrated with sterile deionized (DI) water and incubated in a non‐CO_2_ incubator at 37°C for a minimum of 8 h prior to use. Water was then exchanged for XF calibrant fluid 45–60 min before running the assay. Disc cell spheroids were removed from the agarose microwells, transferred to Seahorse 96‐well spheroid microplates and allowed to rest for at least 1 h prior to running the assay. In brief, spheroid microplates were first coated with 100 μg/mL poly‐D‐lysine (Sigma), rinsed twice with sterile DI water, and air dried for 30 min prior to loading 175 μL of assay media to each well. Complete unbuffered XF assay medium consisted of Seahorse XF DMEM supplemented with 5.5 mM glucose, 1 mM sodium pyruvate, and 2 mM L‐glutamine (all Agilent Technologies). Media was made fresh, pH adjusted to 7.4, and warmed to 37°C in a waterbath. Blank wells (XF assay medium only) were prepared without spheroids to remove background OCR and ECAR during analysis. Extracellular flux measurements were performed six times at 20‐min intervals under basal conditions, that is, no injection of inhibitor/stimulator treatments. Following the Seahorse assay, metabolic rate samples were removed from the 96‐well microplate and transferred into microtubes together with assay media, before then aspirating off the media and storing samples at −80°C until digestion in a papain enzyme solution (60 μL per spheroid) of 100 mM sodium phosphate/5 mM Na_2_EDTA buffer, 3.88 U/mL of papain enzyme and 5 mM L‐cysteine, pH 6.5 (all from Sigma‐Aldrich) at 60°C under constant rotation (10 r.p.m) for 18 h.

Raw measurements of oxygen level (mmHg) and pH, as shown in Figure S5, were extracted from Wave software (Agilent Technologies) and data analysis was performed in Excel. In short, metabolic rates were calculated over the linear/plateaued region of the data set (after 10 min for oxygen) and pH measurements were converted to lactate concentration using a standard curve which had been created using the Seahorse XFe96 analyzer system and the same assay medium containing different and known concentrations of lactate. OCR and LPR calculations were normalized by cell number per well (i.e., per spheroid). The cell number of each spheroid was established using a Quant‐iT PicoGreen dsDNA kit (Thermo Fisher Scientific) and interpolation using a purpose made standard curve for DNA content versus cell number. As ECAR is a measure of glycolysis, glucose consumption rate (GCR) was estimated based on the assumption that ~2 mol of lactate are produced for every mole of glucose consumed by highly glycolytic disc cells.^49^ At least three biological repeats were performed for each species with prep/donor‐matched NP and AF cells (*N* = 3). Metabolic rates were determined for both cell types as they are required as parameters to model an intact IVD, consisting of a separate NP and AF domain, in order to predict full gradients and nutrient distributions throughout the disc. Human cells in this work were isolated from a 33‐year‐old female, a 41‐year‐old female, a 44‐year‐old male, and a 65‐year‐old male (*N* = 4). Technical replicates were analyzed for outliers using the ROUT method. A minimum of 25 replicates passed the outlier test with an average of two outliers removed per dataset.

Meanwhile, only NP cell spheroids were created for the assessment of GAG production since perspective cell‐based therapies need to be characterized by their matrix synthesizing abilities and regeneration in terms of restoring NP matrix. These spheroids were formed as individual cell aggregates in a flat‐bottomed 96‐well plate, which was coated with a thin layer of 2% agarose to prevent cell attachment. Each spheroid was cultured in 200 μL of phenol‐free LG‐DMEM supplemented with 10% FBS and 2% Pen‐Strep (all Gibco), with a media exchange performed every 3 days for 2 weeks (37°C in a humidified atmosphere with 5% CO_2_ and 10% O_2_). Aspirated media was retained at every feed, as on termination of culture the spheroid‐specific media and spheroid sample were combined and stored at −80°C until lyophilization using a standard drying protocol (0.200 mBar, −10°C, 16–18 h). To ensure sufficient GAG accumulation, for detection through a dimethylmethylene blue dye‐binding assay (Blyscan, Biocolor Ltd.), three to five spheroids were pooled per technical replicate (together with their media) and dried samples were subsequently papain digested in 100 μL/spheroid.

### 2.6 Predicting the effect of cell injection on the species‐specific nutrient microenvironment and regeneration timeline

The in silico nutrient transport model was created using COMSOL Multiphysics and the steady‐state nutrient microenvironment was governed by species‐specific metabolically active cell densities and metabolic rates determined. An explanation of the equation coupling, and computational methodology can be found in our previous work, together with successful experimental validation within an ex vivo disc organ culture system.^21^, ^44^ Briefly, experimentally measured OCR was implemented into coupled reaction–diffusion equations dependent on local oxygen and pH concentration, by employing equations derived and published previously^22^, ^24^, ^50^:

(1) $$Q^{O_{2}}=-\frac{V_{\max}\left(\mathrm{pH}-4.95\right)C^{O_{2}}}{K_{m}\left(\mathrm{pH}-4.59\right)+C^{O_{2}}}\rho_{\text{cell}}$$

where $Q^{O_{2}}$ is the consumption rate (μM/h), t is time (h), $C^{O_{2}}$ is the local oxygen concentration (μM), pH is local pH level and $\rho_{\text{cell}}$ is the active cell density (million cells/mm^3^). $V_{\max}$ is the experimentally measured metabolic rate (OCR; nmol/million cells/h) and $K_{m}$ is the rate limiting Michaelis–Menten constant, that is, the oxygen concentration (μM) at which consumption rate is at half of its maximum.^21^ Glycolysis was experimentally measured as LPR; thus an assumption was made of a 2:1 ratio of lactate to glucose, and GCR was extrapolated.^22^, ^24^, ^50^, ^51^ GCR was modeled as being rate limited to local glucose concentration, as described in our previous work^21^:

(2) $$Q^{\text{gluc}}=-\frac{V_{\max}\left(C^{\text{gluc}}\right)}{K_{m}+C^{\text{gluc}}}\rho_{\text{cell}}$$

where $Q^{\text{gluc}}$ is the consumption rate (μM/h), $C^{\text{gluc}}$ is the local glucose concentration (mM). Additional input parameters such as metabolite diffusion coefficients for each tissue domain are presented in Table 1.

**TABLE 1:** Effective diffusion coefficients (mm^2^/h) used in silico for nucleus pulposus (NP) and annulus fibrosus (AF) domains of rat, goat, and human discs.

	Rat		Goat		Human		
	NP	AF	NP	AF	NP	AF
*D*_gluc_	1.22^a^	Axial: 0.7 Radial: 0.52^b^	1.22^a^	Axial: 0.7 Radial: 0.52^b^	1.17^c^	Axial: 0.45 Radial: 0.37^d^
*D*_o2_	5^e^	3.78^f^	5^e^	3.78^f^	4.81^c^	Axial: 3.08 Radial: 2.20^d^
*D*_lac_	1.62^a^	1.2^a^	1.62^a^	1.2^a^	1.56^c^	Axial: 0.61 Radial: 0.50^a^

Abbreviations: D_gluc_, glucose diffusion coefficient; D_lac_, lactate diffusion coefficient; D_o2_, oxygen diffusion coefficient.

^a^ Glucose measurements, lactate derived from glucose.^48^, ^74^, ^113^

^b^ Experimentally determined in axial and radial direction for bovine tissue under 10% strain.^114^

^c^ Experimentally measured in the literature and hydration adjusted as per our previous work.^48^

^d^ Literature values for human tissue under 10% strain and temperature adjusted as described previously.^48^

^e^ Theoretical values from literature 17.

^f^ Experimentally determined axially for bovine tissue under 15% strain.^115^

A transient GAG regeneration model was created based on the conservation of mass for GAG theory established previously by Gu et al.^52^ for human IVD^53^:

(3) $$\frac{\partial \left(c^{\mathrm{GAG}}\right)}{\partial t}+\nabla \left(c^{\mathrm{GAG}}v^{s}\right)=Q^{\mathrm{GAG}}$$

where $c^{\mathrm{GAG}}$ is the molar concentration of GAG (per tissue volume) and $v^{s}$ is the velocity of solid matrix. $Q^{\mathrm{GAG}}$ is the synthesis/degeneration rate of GAG (per tissue volume), that is, the rate of GAG content change and was simply modeled in this work as the balance between GAG synthesis rate ($Q^{\mathrm{syn}}$) and GAG degradation rate ($Q^{\deg}$):

(4) $$Q^{\mathrm{GAG}}=Q^{\mathrm{syn}}-Q^{\deg}$$

GAG synthesis rate was assumed to be dependent on the viable cell density, $\rho^{\text{cell}}$, and GAG degradation rate is proportionally related to local GAG content ($c^{\mathrm{GAG}}$):

(5) $$Q^{\mathrm{GAG}}=\lambda_{1}\rho^{\text{cell}}-\lambda_{2}c^{\mathrm{GAG}}$$

where $\lambda_{1}$ is the experimentally measured GAG synthesis rate per cell (species‐specific) and $\lambda_{2}$ is the GAG degradation rate. Under the condition of a healthy disc, degeneration does not occur and as a result $Q^{\mathrm{GAG}}$ is assumed to be zero. Therefore, the above equation becomes:

(6) $$\lambda_{1}=\frac{\lambda_{2}{c_{0}}^{\mathrm{GAG}}}{{\rho_{0}}^{\text{cell}}}$$

where ${c_{0}}^{\mathrm{GAG}}$ is the GAG content prior to degeneration and ${\rho_{0}}^{\text{cell}}$ is the viable cell density at a healthy state. The GAG degeneration rate ($\lambda_{2}$) was assumed to remain constant and has previously been calculated based on the half‐life of GAG^52^, ^53^:

(7) $$\lambda_{2}=\frac{\ln2}{\tau }$$

where τ is the half‐life of GAG turnover and has been reported to be 11 years.^54^ This value has been used in a previous model where predictions were consistent with measured results.^52^, ^54^, ^55^

Species‐specific GAG content was derived from literature and is presented in Table 2.^15^, ^16^, ^56^ For human, the initial value set in the model is a disc with Grade III degeneration deemed suitable for treatment with cell injection. For animal models, native healthy GAG content was adjusted according to qualitative histological staining following puncture injury or chondroitinase ABC (chABC) injection for rat and goat, respectively. In rat, this corresponded to a 100% reduction in GAG based on Safranin‐O staining by Barcellona et al.^38^ for caudal discs punctured with a 27‐G needle and left 2 weeks prior to treatment. In goat, this corresponded to a 50% reduction in GAG based on AB staining of lumbar discs injected with 1 U of chABC and left 12 weeks prior to treatment.^57^ Similarly according to literature, it can be assumed that establishing an injury model significantly disrupts cellularity. As a result, a 75% and a 50% reduction in cellularity was employed for rat and goat, respectively.^58^, ^59^ Furthermore, μg/mg dry weight (DW) of GAG was converted to μg/tissue volume for input into the in silico model using a species‐specific mg DW/mm^3^ conversion factor which was determined experimentally.

**TABLE 2:** Glycosaminoglycan (GAG) parameters used in silico for nucleus pulposus (NP) and annulus fibrosus (AF) domains of rat, goat, and human discs.

	Rat		Goat		Human	
	NP	AF	NP	AF	NP	AF
Healthy/native GAG content (μg/mg DW)	95.2^a^	37.5^b^	335^c^	95^b^	537.8^d^	153^d^
Degenerated GAG content (μg/mm^3^)	0	10.6	47.4	26.9	80.7^e^	30.3^e^
GAG synthesis rate (pg/cell/day)	6.19^f^	—	9.08^f^	—	8.48^f^	—

Abbreviation: DW, dry weight.

^a^ Cd9‐10 of 12‐month male Sprague Dawley rats.^15^

^b^ Averaged for inner and outer AF.^15^, ^16^

^c^ L4‐5 of 2.5–3.5 year male goats.^16^

^d^ Grade II human tissue, AF averaged over inner/outer and anterior/posterior region.^56^

^e^ Grade III human tissue, AF averaged over inner/outer and anterior/posterior region.^56^

^f^ Experimentally determined in this study.

### 2.7 Towards experimental validation of in silico nutrient transport models

Uncertainty remains regarding metabolite concentrations surrounding the disc boundary, particularly for rat caudal and goat lumbar discs. Similar to previous numerical models, it was assumed that glucose concentrations and pH levels at the periannular boundary are that of blood plasma in the surrounding blood vessels.^60^, ^61^, ^62^ Concentrations at the NP boundary, underneath the endplate, were estimated based on the reduction in concentration through the cartilaginous endplate modeled in our previous work.^48^ Oxygen values from <1% O_2_ to 6% O_2_ have been used as boundary conditions throughout the literature.^63^ In an attempt to refine boundary concentrations and improve confidence, models were first run for “healthy” conditions, with native cell densities and geometries. Healthy or uninjured rat caudal discs and goat lumbar discs were then probed experimentally using our previously established methods.^44^ Briefly, intradiscal oxygen and pH values were measured using PreSens probes (Regensburg, Germany) and glucose was assessed biochemically using an enzymatic‐colorimetric assay (Sentinel Diagnostics, Italy). These experimentally measured values for oxygen and pH where then compared with predicted profiles in order to iteratively determine the boundary concentrations and enhance confidence in the modeling capabilities. Finalized boundary concentrations are presented in Table S1.

### 2.8 Statistical analysis

One‐way ANOVA was used for analysis of variance using GraphPad Prism (version 10) software. Tukey's multiple comparison test was used to compare between groups. Results are displayed as mean ± SD, where n represents the number of biological replicates. Significance was accepted at a level of *p* < 0.05.

## 3 RESULTS

### 3.1 Experimentally determined species‐specific parameters

**FIGURE 3:**
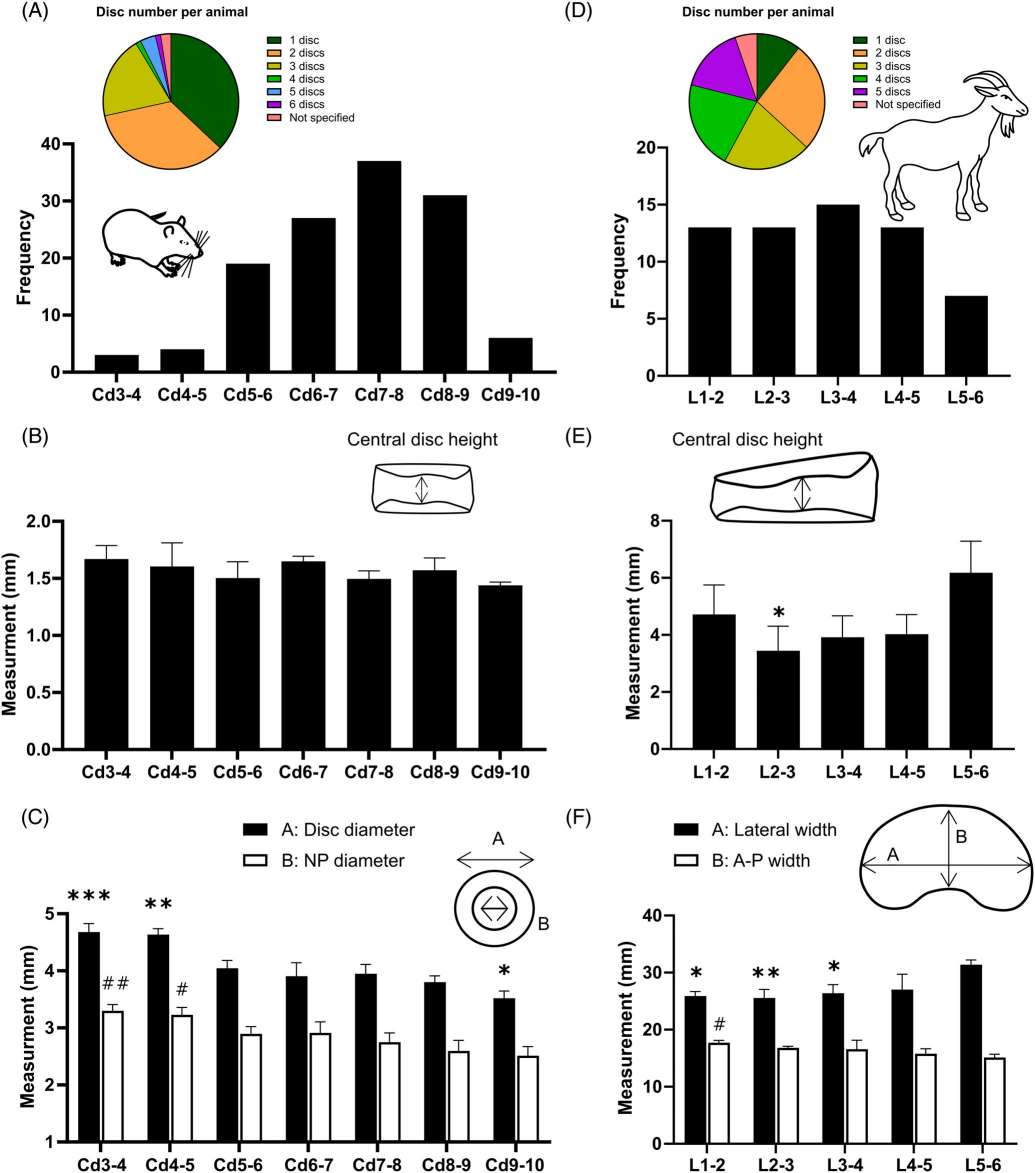
Geometrical analysis of rat caudal level Cd3‐4 to Cd9‐10 and goat lumbar level L1‐2 to L5‐6. (A) Literature search results for the number of discs per animal and frequency of caudal levels used in published rat tail studies. (B) Experimentally measured central disc height for 8‐week‐old Wistar rats (*N* = 6), with no statistical significance found between Cd7‐8 (most frequently used) and all other levels within this range. (C) Corresponding external disc diameter and internal nucleus pulposus (NP) diameter. Statistics indicate a significant difference to Cd7‐8 full disc (*) and NP (#) diameter with *p* < 0.05. (D) Literature search results for the number of discs per animal and frequency of lumbar levels used in published goat studies. (E) Experimentally measured central disc height for skeletally mature Saanen goats (*N* = 3), a significant difference was only found between L2‐3 and L5‐6 (least frequently used). (F) Corresponding lateral and anterior to posterior (A‐P) width. Statistics indicate a significant difference to L5‐6 lateral (*) and A‐P (#) width.

To compare the IVD scale‐effect between species, the geometry of rat and goat discs was first assessed in relation to their caudal and lumbar level, respectively. From Figure 3A it appears that only one or two caudal discs are used per rat and there is a strong inclination towards using levels Cd6‐7 to Cd8‐9, with ~30% of studies using Cd7‐8. Importantly, Cd7‐8 was found to be not significantly different from all other investigated levels in terms of disc height (Figure 3B). Additionally, only discs outside the commonly used range (Cd6‐7 to Cd8‐9) had a disc diameter significantly larger (Cd3‐4 at *p* = 0.0007 and Cd4‐5 at *p* = 0.0012) and smaller (Cd9‐10 at *p* = 0.0467) than Cd7‐8, Figure 3C. Similarly, only Cd3‐4 (*p* = 0.0096) and Cd4‐5 (*p* = 0.0259) had a NP diameter significantly larger than Cd7‐8. For the goat models in Figure 3D, it appears less common to use only one level per animal than to use all five lumbar discs, with no clear predominant level of choice. Despite this, L5‐6 is selected half as frequently as L1‐2 to L4‐5 and although disc height appears to vary down the lumbar spine, only the central disc height of L2‐3 was found to be significantly different to L5‐6 (*p* = 0.0255), Figure 3E. Results in Figure 3F show that as discs progress down the lumbar section they widen laterally, and the anterior to posterior (A‐P) width shortens. Lateral width of L5‐6 was significantly larger than L1‐2 (*p* = 0.0137), L2‐3 (*p* = 0.0094) and L4‐5 (*p* = 0.0240), while only the A‐P width of L1‐2 was significantly larger than L5‐6 (*p* = 0.0257).

**FIGURE 4:**
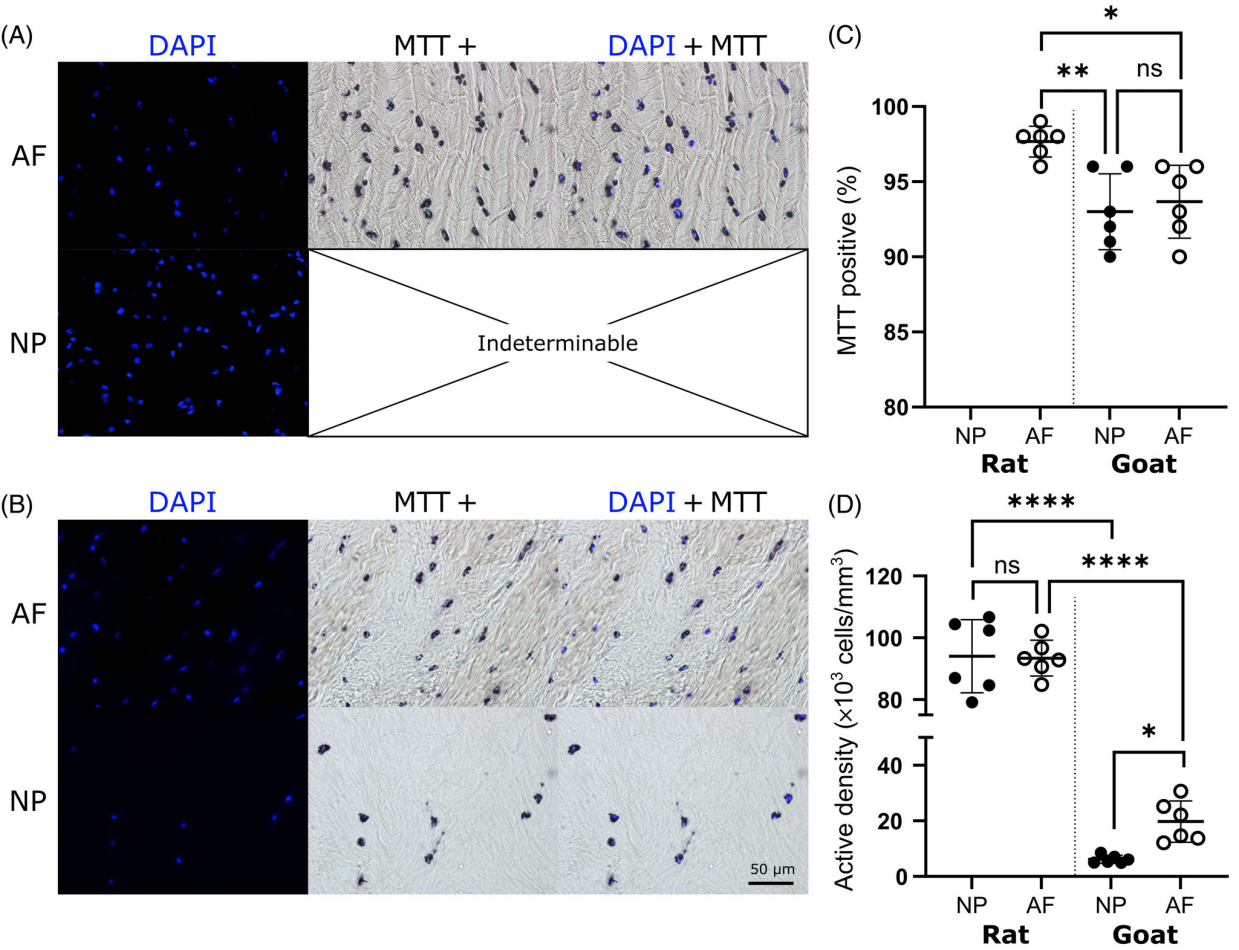
Experimentally determined metabolically active cell density for the nucleus pulposus (NP) and annulus fibrosus (AF) of rat caudal and goat lumbar discs. (A) Native rat and (B) goat tissue fluorescently 4′,6‐diamidino‐2‐phenylindole (DAPI) stained to indicate the nuclei of all cells and methylthiazolyldiphenyl‐tetrazolium bromide (MTT) brightfield imaged to identify colocalized formazan crystal deposition around metabolically active cells. (C) Percentage of cells quantified as MTT positive, with rat AF tissue determined as significantly higher than goat AF (*p* = 0.013) and NP (*p* = 0.004) while no significant difference was determined between goat AF and NP (*p* = 0.849). MTT+ visualization within rat NP was not feasible due to the highly gelatinous composition. (D) Species‐specific metabolically active cell density, assuming a similar percentage of MTT positive cells in rat NP as determined in the AF. No significance was found between rat NP and AF cell density (*p* = 0.999), while goat AF had a significantly higher cell density than NP (*p* = 0.026). Additionally, it was determined that rat tissue has a significantly greater cell population than the corresponding region of goat tissue (*p* < 0.0001).

Figure 4A presents the native cell density of rat tissue, with separate AF and NP regions fluorescently stained with DAPI, while MTT brightfield images, showing colocalized formazan crystals, is only presented for AF, as it was indeterminable in the NP matrix due to technical challenges owing to its highly gelatinous nature and inability to retain the formazan crystals. Similarly, Figure 4B presents native cell density of goat tissue, with a clear distinction between the fibrous lamella of the AF and sparser cell distribution in the NP matrix. Figure 4C presents the percentage of cells quantified as MTT positive, with rat AF tissue determined as significantly higher than goat AF (*p* = 0.013) and NP (*p* = 0.004) while no significant difference was determined between goat AF and NP (*p* = 0.849). MTT+ visualization within rat NP was not feasible due to the highly gelatinous composition. Figure 4D presents the quantified species‐specific metabolically active cell density. It was assumed that the percentage of MTT positive cells in rat NP would be similar to AF, as no significant difference was determined between NP and AF in goat. No significance was found between the cell density of rat NP (94 × 10^3^ cells/mm^3^) and AF (93 × 10^3^ cells/mm^3^) tissue (*p* = 0.999), while goat AF (16 × 10^3^ cells/mm^3^) had a significantly higher cell density than NP (6 × 10^3^ cells/mm^3^; *p* = 0.026). Additionally, it was determined that rat tissue has a significantly greater cell population than the corresponding region of goat tissue (*p* < 0.0001).

**FIGURE 5:**
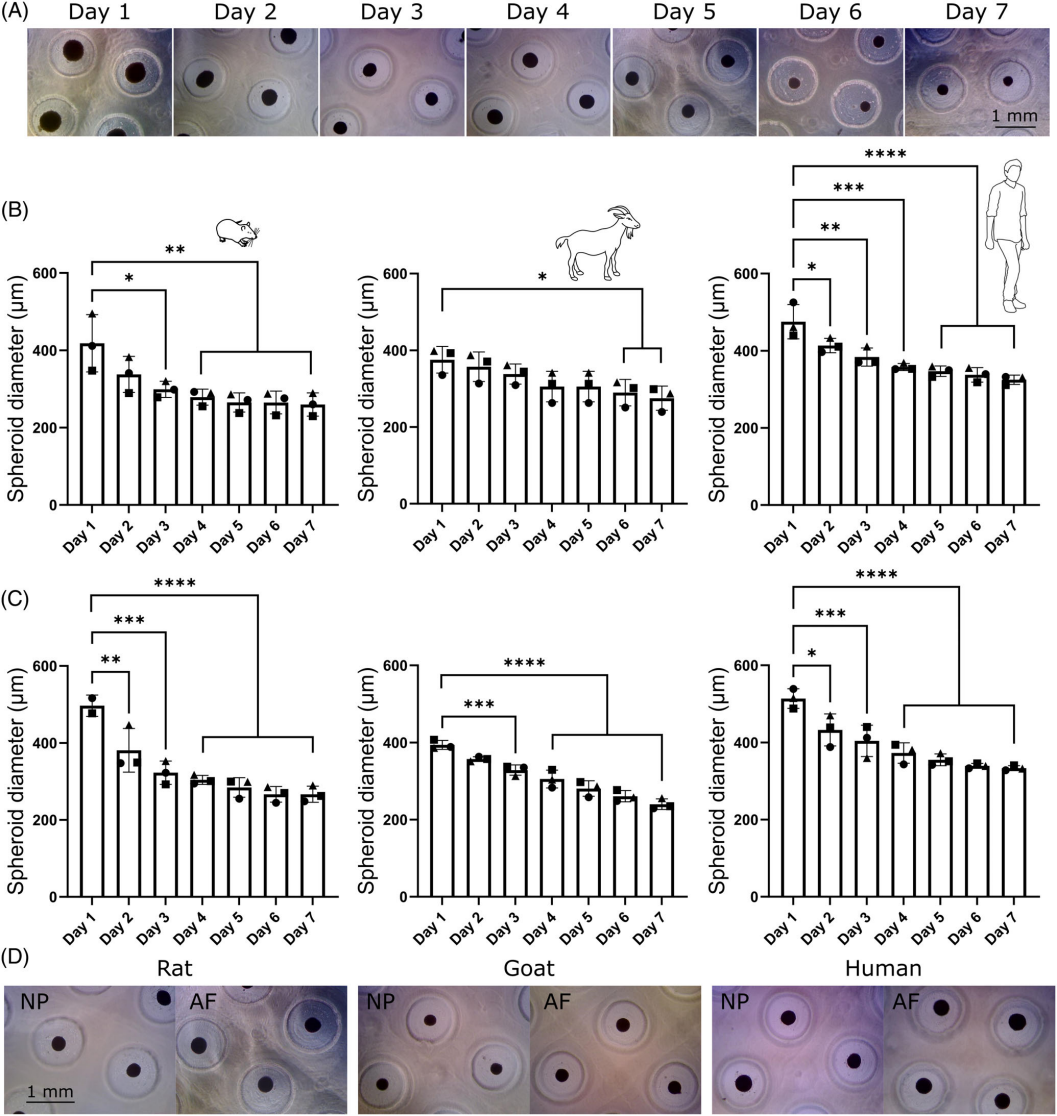
Temporal assessment of disc spheroids (nucleus pulposus [NP] and annulus fibrosus [AF]) from rat, goat, and human over a 7‐day culture period. (A) Example of daily microscopic images showing rat NP spheroids within agarose microwells. (B) Quantification of spheroid diameters for rat, goat, and human NP cells. Rat spheroids became statistically different to Day 1 at Day 3 (*p* = 0.012), goat at Day 6 (*p* = 0.045), and human at Day 2 (*p* = 0.024). (C) Quantification of spheroid diameters for rat, goat, and human AF cells. Rat spheroids became statistically different to Day 1 at Day 2 (*p* = 0.005), goat at Day 3 (*p* = 0.0007), and human at Day 2 (*p* = 0.0101). (D) Microscopic images of both NP and AF spheroids from rat, goat, and human after 7 days.

Species‐specific disc spheroids were assessed daily in order to ensure that both cell types (NP and AF) from each species (rat, goat, and human) were capable of forming stable cellular aggregates within the designed culture system over a 7‐day period. Figure 5A consists of representative daily microscopic images showing rat NP spheroids within the agarose microwell array. Figure 5B,C presents diameters for rat, goat, and human NP and AF spheroids, respectively. Spheroid diameters were quantified daily to assess condensation and identify when stable spheroids are formed based on the plateau of the temporal graph. Results showed no significant difference in diameters between consecutive days, except for Days 1 and 2 in rat AF, human NP, and human AF. In general, all spheroids condensate with diameters decreasing over the first few days. Rat and human spheroids plateaued fastest with diameters becoming consistent on Days 4–5, whereas goat spheroids continued to condensate up to Days 6–7. Figure 5D consists of representative microscopic images of both NP and AF spheroids from rat, goat, and human after 7 days.

**FIGURE 6:**
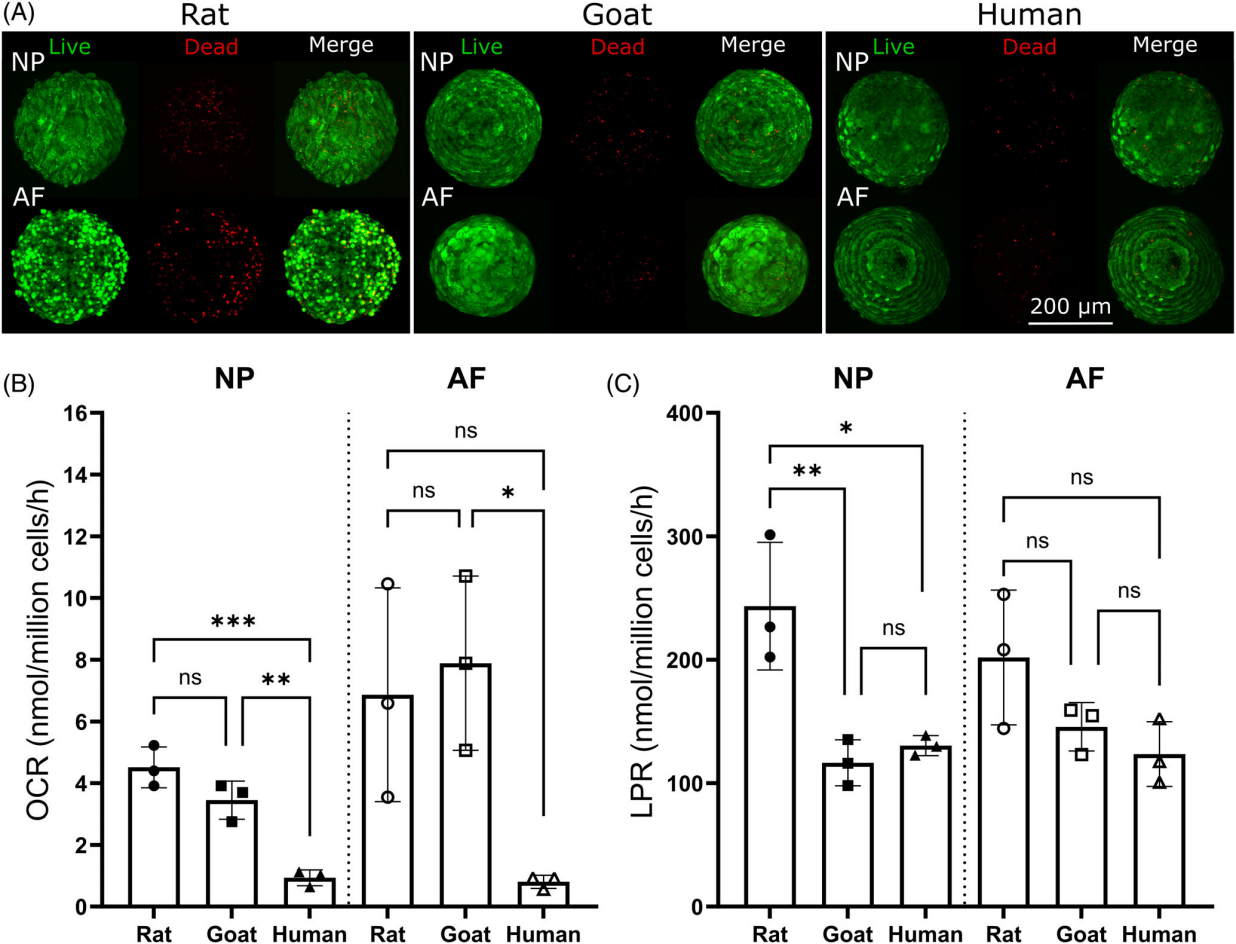
Viability assessment and measured metabolic rates of nucleus pulposus (NP) and annulus fibrosus (AF) spheroids from rat, goat, and human. (A) Spheroids were assessed using Live/Dead staining to ensure viability remained high prior to carrying out metabolic rate measurements. (B) Oxygen consumption rates (OCR) and (C) lactate production rates (LPR) for disc cells assessed in a 3D spheroid configuration (*N* = 3). Human NP cells had a significantly lower OCR than rat NP (*p* = 0.0005) and goat NP (*p* = 0.0031), while human AF was only significantly lower than goat AF (p = 0.0352). Rat NP cells had a significantly higher LPR than goat NP (*p* = 0.0068) and human NP (*p* = 0.0119), while no significant differences were detected for AF cells.

Prior to carrying out metabolic flux analysis, spheroid viability was qualitatively assessed to ensure an acceptable level of cell viability (>80%) to later perform normalization of consumption and production rates. Due to the 3D and compact cell nature of spheroids, an exact quantitative assessment was not feasible. Figure 6A presents representative images indicating a high viability of both NP and AF spheroids for all species within agarose microwells. Figure 6B shows that in 3D spheroid form, human NP cells have a significantly lower OCR than rat NP cells (*p* = 0.0005) and goat NP cells (*p* = 0.0031), while human AF was significantly lower than goat AF (*p* = 0.0352). Furthermore, Figure 6C shows that rat NP cells had a significantly higher LPR than both goat NP cells (*p* = 0.0068) and human NP cells (*p* = 0.0119), while no significant differences were detected among AF cells across the different species.

**FIGURE 7:**
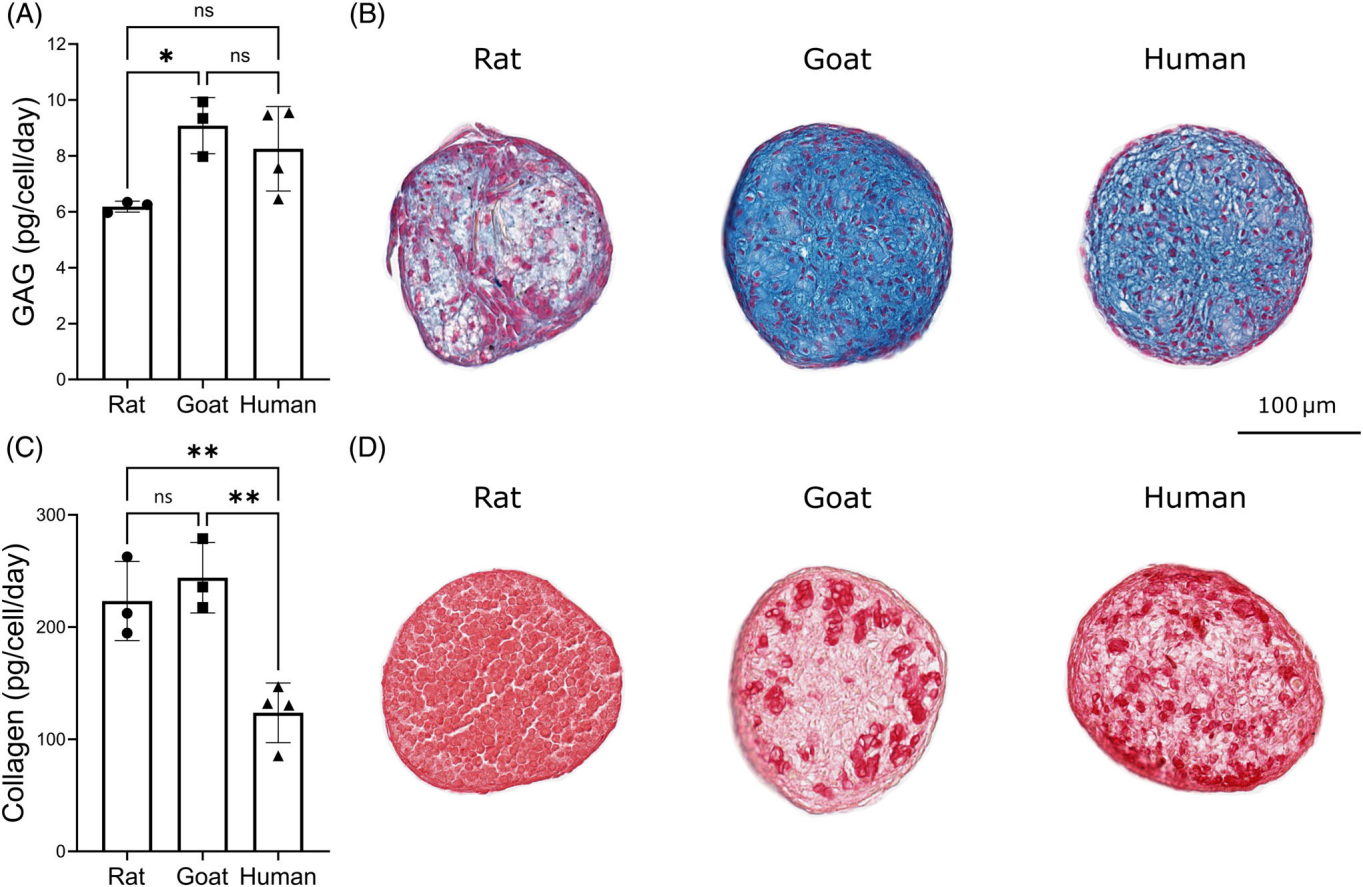
Species‐specific matrix synthesis rates and histological evaluation of nucleus pulposus (NP) micro‐spheroids. (A) Glycosaminoglycan (GAG) production rates for rat (*N* = 3), goat (*N* = 3), and human NP cells (*N* = 4) over a 2‐week period. Rat NP cells had a significantly lower production rate than goat NP cells (*p* = 0.0385). (B) Corresponding histological evaluation using alcian blue (AB) to stain for GAG. (C) Collagen production rates for rat (*N* = 3), goat (*N* = 3), and human NP cells (*N* = 4) over a 2‐week period. Collagen production rates for human NP cells were significantly lower than both rat (*p* = 0.0093) and goat (*p* = 0.0033) NP cells. (D) Corresponding histological evaluation using picrosirius red (PSR) to stain for collagen.

Figure 7 presents species‐specific matrix synthesis rates calculated over a 2‐week culture period and normalized by DNA content/cell number per microspheroid. Furthermore, histological evaluation is included, highlighting GAG and collagen distribution within the microspheroids using AB and PSR staining, respectively. GAG synthesis rates of rat NP cells were found to be significantly lower than goat NP cells (*p* = 0.0385). However, no significant difference was detected between human NP cells and the two animal species. Nonetheless, human NP cells had significantly lower collagen synthesis rates than both rat (*p* = 0.0093) and goat (*p* = 0.0033) NP cells, while no significant difference was detected between the two animal species.

### 3.2 Predicted differences in GAG regeneration capacity and nutrient microenvironments between species

**FIGURE 8:**
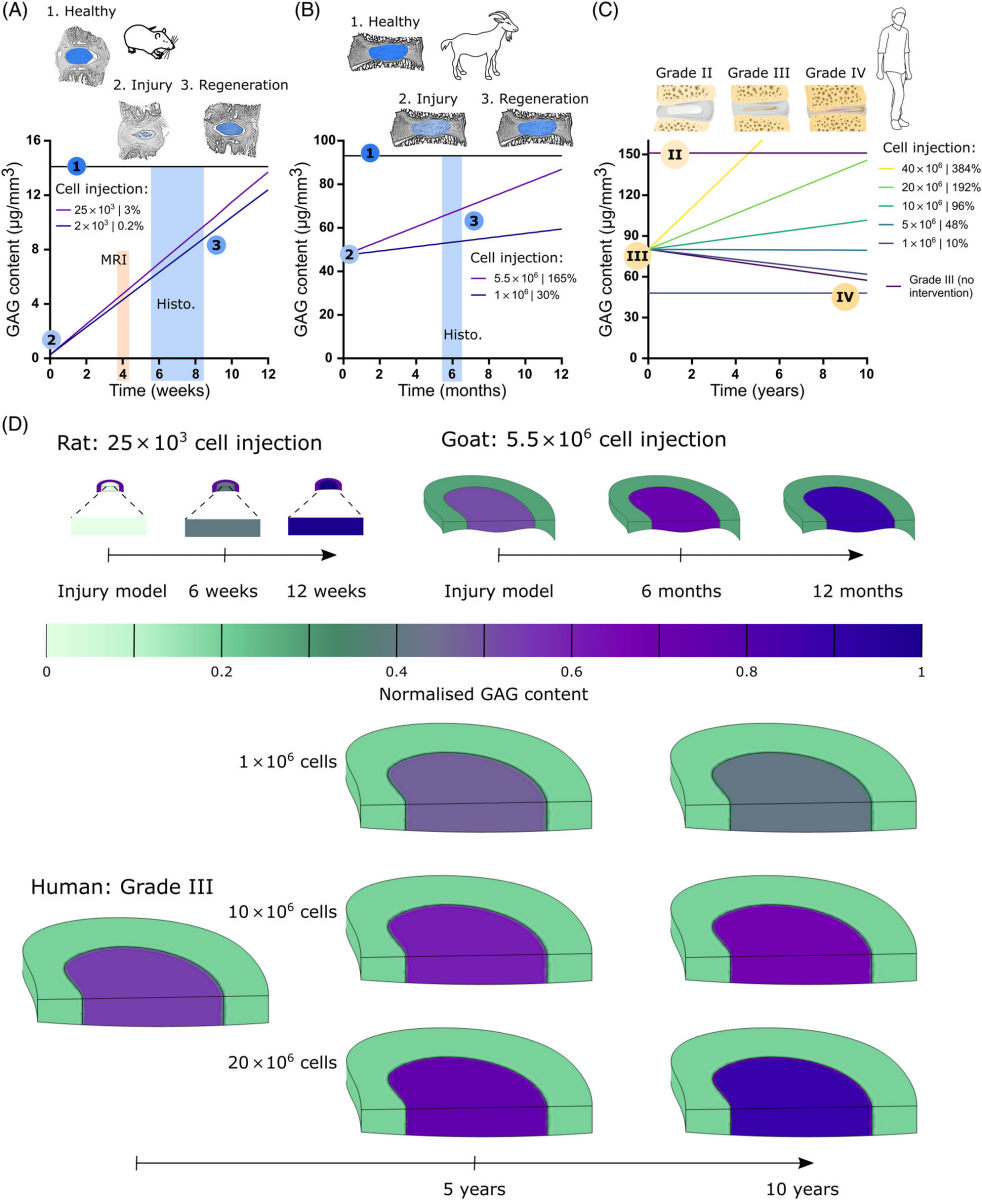
Predicted glycosaminoglycan (GAG) matrix regeneration in the nucleus pulposus (NP) of a pre‐clinical rat model, goat model and human clinical trials for cell‐based therapies. (A) Predicted GAG regeneration in a rat degeneration model injected with 2 × 10^3^ (0.2% of healthy NP cell population) or 25 × 10^3^ (3% of healthy NP cell population) cells over a 12‐week study. The shaded regions either indicate when MRI signal has been reported as significantly higher or histological GAG staining significantly stronger compared with an injured control, in the literature. (B) Predicted GAG regeneration in a goat degeneration model injected with 1 × 10^6^ (30% of healthy NP cell population) or 5.5 × 10^6^ (165% of healthy NP cell population) cells over a 12‐month study. (C) Predicted GAG regeneration in a clinical trial injected with 1 × 10^6^–40 × 10^6^ cells (10%–383% of Grade III NP cell population). (D) A sample of corresponding contour plots for in silico rat (25 × 10^3^ cells), goat (5.5 × 10^6^ cells), and human models of GAG regeneration (geometries presented to scale). GAG is normalized to native/healthy NP content for rat and goat and Grade II content for human.

Figure 8 presents predicted GAG matrix regeneration in the NP of a preclinical rat and goat model compared with predicted regeneration within human clinical trials for cell‐based therapies. As detailed previously, all investigated parameters were selected based on what has been reported in literature for these specific animal models or registered publicly for clinical trials. Figure 8A predicts that based on the experimentally determined rat matrix synthesis rate, substantial regeneration is feasible in rat caudal models within a 12‐week timeframe. Injection delivery of 2000 or 25 000 cells accounts for only 0.2% or 3% of the healthy species‐specific NP cell density, respectively. Despite these relatively low cell treatment numbers, literature has reported MRI signal to be significantly higher than punctured controls as early as 4 weeks and superior histological staining for GAG at around 6–8 weeks, as highlighted in the graph (Figure 8A).^38^, ^39^, ^40^, ^41^ Figure 8B predicts that based on experimentally determined goat matrix synthesis rates, substantial GAG regeneration is feasible in goat lumbar models within a 12‐month timeframe. Injecting 1 million or 5.5 million cells accounts for 30% or 165% of the healthy species‐specific NP cell density, respectively. It has been reported in literature that cell treatments showed significantly higher aggrecan expression and histological staining compared with injured control and performed superior to acellular treatments around 6 months, as highlighted in the graph.^64^

Figure 8C predicts that based on experimentally determined human matrix synthesis rates, together with the substantially larger scale of the human IVD, the timeframe for functional GAG regeneration is a matter of years and is highly dependent on the number of cells injected clinically. Without cell injection, degeneration is predicted to continue from Grade III towards Grade IV over 10 years. A low dose of 1 million cells fails to prevent further degeneration, while a dose of 5 million cells is predicted to maintain GAG levels at those of Grade III, without further degeneration. A treatment dose of 10 million cells is predicted to be capable of initiating regeneration. Nonetheless, GAG matrix recovery is predicted to be only 65% of Grade II levels after 10 years. Doubling this dose to 20 million cells predicts recovery to Grade II levels of GAG at ~10 years, while quadrupling the dose to 40 million predicts recovery to Grade II levels of GAG within 4 years. Figure 8D presents an example of the in silico contour plots for rat (25 × 10^3^ cells), goat (5.5 × 10^6^ cells), and human models of GAG regeneration. Predicted GAG results are normalized to native/healthy NP content for rat and goat and Grade II content for human. Geometries are presented to scale to emphasize this scale‐effect and the timeframe predicted for animal model regeneration compared with the trajectory of human clinical trials.

**FIGURE 9:**
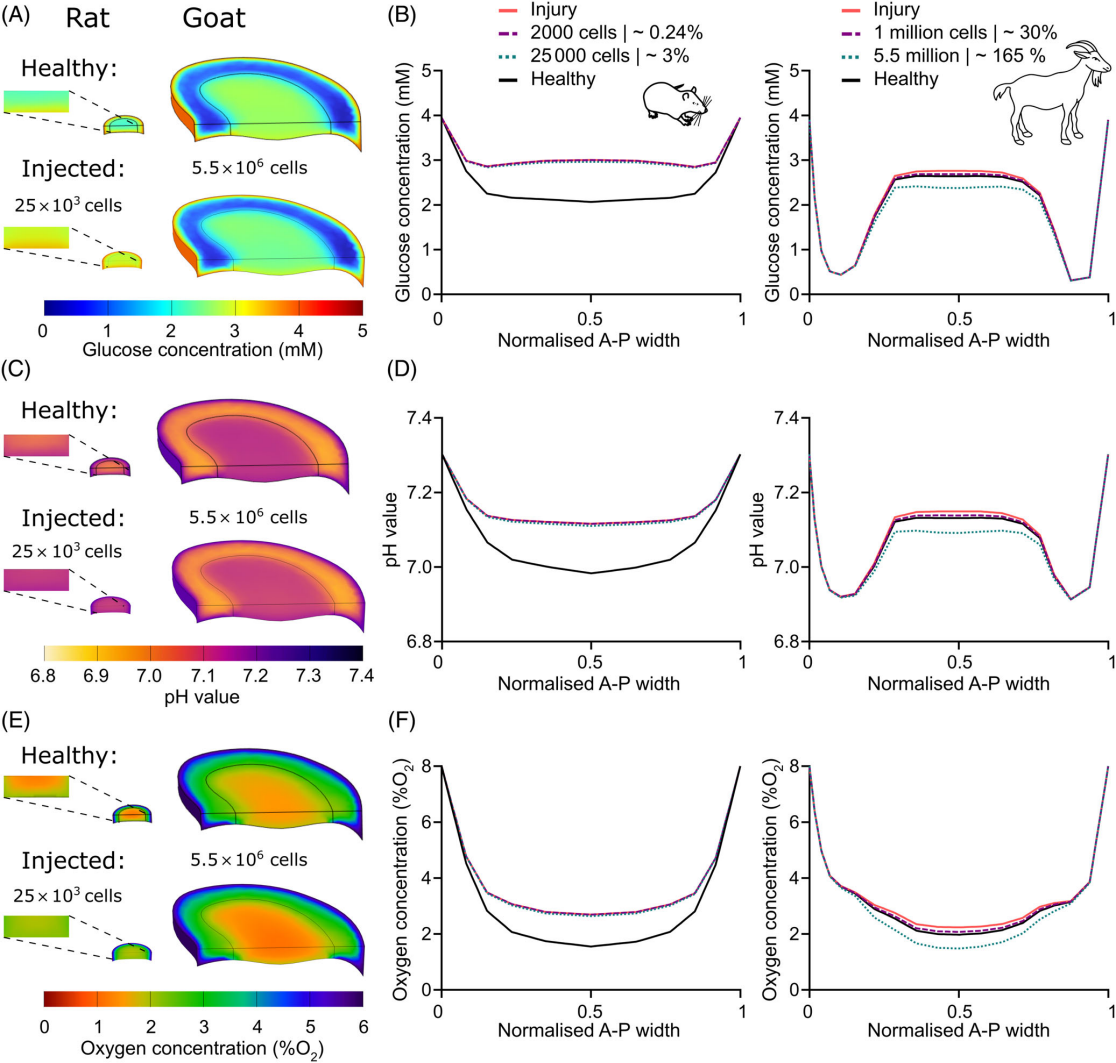
Predicted nutrient microenvironments within pre‐clinical rat and goat animal models assessing cell‐based therapies. (A) Predicted glucose distribution across a healthy rat and goat disc compared with an injury model injected with cells. (B) Anterior to posterior (A‐P) profile for glucose, at midheight, through the corresponding in silico models for rat and goat. (C) Predicted pH distribution across a healthy rat and goat disc compared with an injury model injected with cells. (D) A‐P profile for pH, at midheight, through the corresponding in silico models for rat and goat. (E) Predicted oxygen distribution across a healthy rat and goat disc compared with an injury model injected with cells. (F) A‐P profile for oxygen, at midheight, through the corresponding in silico models for rat and goat.

Figure 9 presents nutrient microenvironments predicted within preclinical rat and goat models. Figure 9A highlights predicted glucose distribution within a healthy rat and goat disc compared with an injury model injected with an upper range of 25 000 and 5.5 million cells, respectively. Figure 9B presents the A‐P profile of glucose through the disc, further incorporating the lower range of cell injection and the effect of injury (without cell injection). Similarly, Figure 9C highlights the predicted pH distribution, Figure 9D the corresponding A‐P profile of pH, Figure 9E highlights predicted oxygen distribution and Figure 9F the corresponding A‐P profile of oxygen through the disc. In summary, it is predicted that a Cd7‐8 disc in rat has an approximate central microenvironment of 2 mM glucose, pH of 6.9 and oxygen level of 1.5% O_2_, while a L3‐4 disc in goat has an approximate central microenvironment of 1.4 mM glucose, pH of 6.9 and oxygen level of 2% O_2_. In both animal models, inducing an injury results in disrupted cell density within the NP and thus predicts higher levels of nutrients and less acid build‐up than a fully functioning healthy disc. Injecting either 2000 or 25 000 cells into an injured rat model is not predicted to alter the injured microenvironment as these cell numbers represent only 0.2% and 3% of the native/healthy NP cell population. However, injecting 1 million cells (30% of native/healthy NP cell population) into the injured goat model is predicted to restore the nutrient microenvironment to that of a healthy state, while injecting 5.5 million cells (165% of native/healthy NP cell population) is predicted to slightly reduce nutrient concentrations and pH below the healthy levels. Results of the partial experimental validation of nutrient transport models, in an undisturbed healthy rat and goat disc are presented in Figure S6 and help to provide confidence in the modeling capabilities.

**FIGURE 10:**
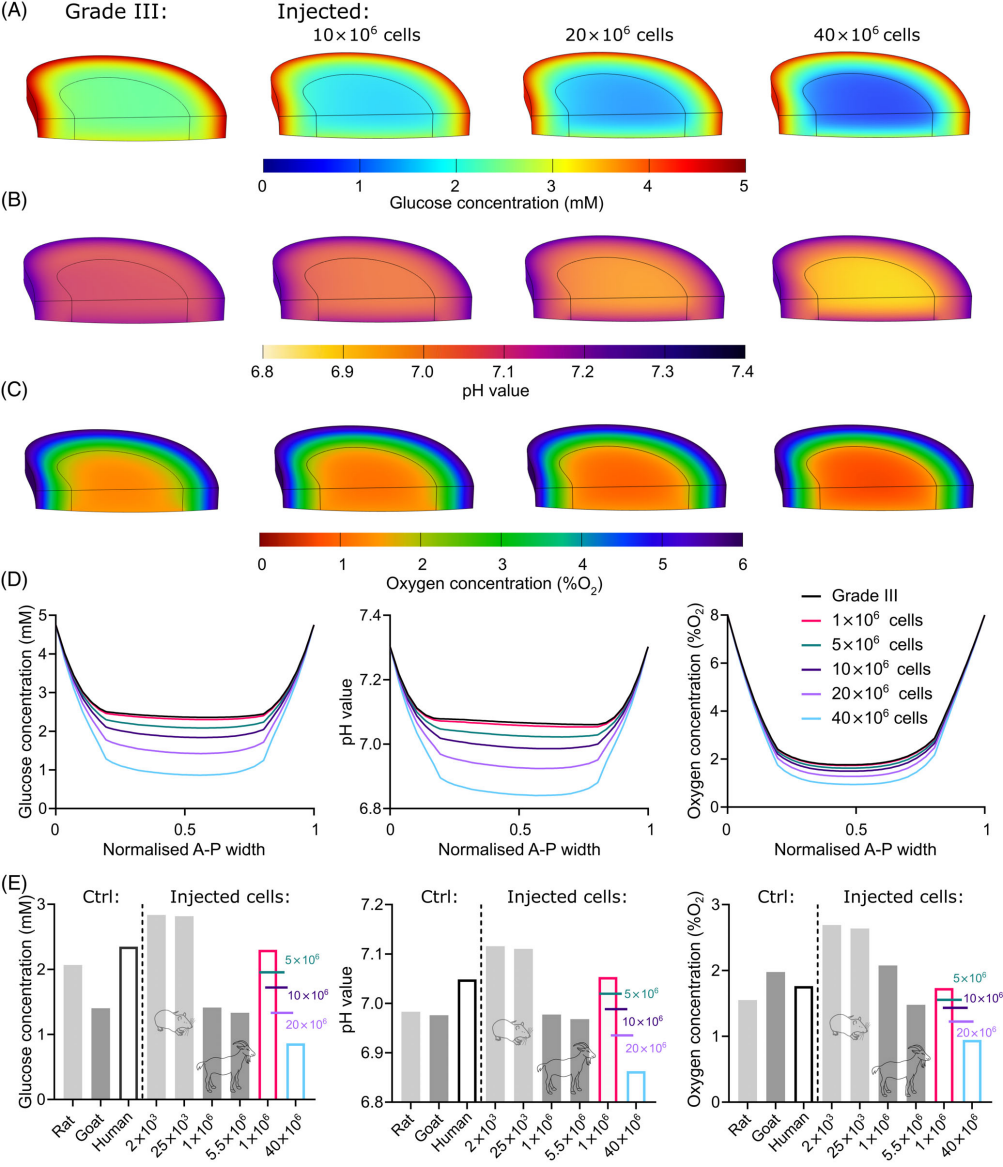
Predicted nutrient microenvironments within a Grade III human intervertebral disc (IVD) undergoing clinical assessment for a range of injected cell numbers. (A) Predicted glucose, (B) pH, and (C) oxygen distribution across a Grade III human IVD with no treatment or with an injection of 10 × 10^6^, 20 × 10^6^, or 40 × 10^6^ cells. (D) Corresponding anterior to posterior (A‐P) profile of glucose, pH, and oxygen at midheight, through each of the in silico models. (E) Minimum metabolite (glucose, pH and oxygen) concentrations within the NP for both preclinical animal models and clinical human models, under control (ctrl) conditions that is, healthy animal and Grade III human, compared with investigated ranges of cell numbers for each species.

Figure 10 presents the predicted nutrient microenvironment within a Grade III human IVD undergoing clinical assessment for a range of injected cell numbers. First, Figure 10A–C presents the distribution of glucose, pH and oxygen across the IVD with either no cell treatment or a high cell dose of 10, 20, or 40 million cells and Figure 10D presents the corresponding A‐P profile of glucose, oxygen and pH for all investigated doses from 1 to 40 million cells. As expected, increasing cell dose further exacerbates the nutrient microenvironment. Second, Figure 10E compares the resulting central NP concentrations across preclinical animal models and clinical human models. An important finding is that the typical ranges of cell injection in rat and goat models do not appear to significantly alter the nutrient microenvironment from its healthy steady‐state levels. However, different nutrient microenvironments are predicted to be established clinically depending on the number of cells being implanted. For example, at the highest dose of 40 million cells, glucose is predicted to drop below 1 mM, pH to 6.8 and oxygen below 1% O_2_.

## 4 DISCUSSION

A significant hurdle for potential cell‐based therapies is the subsequent survival and regenerative capacity of implanted cells.^22^, ^29^, ^65^, ^66^, ^67^ While many exciting developments have demonstrated promise in this regard preclinically, cell‐based therapies for IVD degeneration fail to translate equivalent clinical efficacy.^5^, ^68^, ^69^, ^70^ As a result, an urgency to ascertain the clinical relevance of different animal models has not only ethical merit but also important scientific value for accelerated and successful translation. This work investigated both a small and large animal model, comparing them to the human IVD from the perspective of anatomical scale, cellular metabolism and regenerative potential. Importantly, this work highlights the power of in silico modeling, not only to predict the regeneration timeframe of animal models compared with the more stunted success of human clinical trials, but to inform the design of preclinical assessment and manage the expectations of clinical evaluation.

With regard to lower back pain, there is a clinical consensus that short‐term pain should be treated conservatively. However, when interventions are considered to combat degeneration, a therapy would ideally need to be minimally invasive, delivered in a single operation and as easily adopted by clinicians as standard operating procedures such as microdiscectomy. Given that the degenerative cascade has been characterized by a loss of viable and functional cells, regenerative medicine or tissue engineering approaches propose minimally invasively replenishing the IVD with an injection of active cells. The overall objective is to restore tissue structure and preserve spinal motion. As a result, implanted cells must produce a sufficient amount of disc‐specific ECM and inhibit or retard further degeneration in vivo. While no animal studies completely restore the IVD structure, preclinical results suggest cause for optimism.^3^ However, the effect of a challenging biochemical microenvironment on viability and normal cell function will undoubtedly impact the degree of subsequent regeneration. Therefore, it is imperative to determine (i) how many cells are needed to restore the NP matrix, (ii) in what timeframe functional results are to be expected, (iii) how this may vary across species, and (iv) whether the microenvironment can sustain such a cell dose without further exacerbating the challenging biochemical niche.

Based on preclinical literature there is a strong preference for the rat caudal model over a large animal model such as goat, both from the perspective of feasibility and affordability. The lower financial and animal husbandry burden is reflected in the high number of rats per study and an inclination towards using only one or two caudal levels per animal, as opposed to fewer goats and the use of almost all lumbar levels per study.^41^, ^57^, ^71^, ^72^ According to the geometrical analysis there is no major concern that caudal level could be a confounding factor on the nutrient microenvironment between Cd5 and Cd9, as no significant difference in geometry was identified. As a consequence, it may be advised to remain within this most commonly used range. Similarly, goat lumbar level geometry is not significantly different between L1 and L5. However, L5‐6 should be considered different to other lumbar levels in terms of disc height and width (both lateral and A‐P). The profound importance of disc geometry and scale comes into its own when comparing species. For example, it is estimated that a single human NP is almost 800 times the volume of a rat NP and 12 times that of a goat NP. Furthermore, this study highlights the astronomically higher cell density of the smaller animal models, with rat having a 60‐fold higher and goat a 4‐fold higher NP cell density than the sparse human densities measured previously.^73^, ^74^ As mentioned earlier, it is often hypothesized that the diffusion distances across a disc will determine central metabolite concentrations along with the cellular density, with an inverse relationship between disc height and cell density.^19^ This is reflected throughout the current work with the scale‐effect on both geometry and cell density balancing out to establish relatively similar nutrient concentrations within healthy rat, goat, and human discs. Although no other study has attempted to quantify and compare the metabolically active cell density of both NP and AF tissue across different species, the values can be compared with individual rat and goat studies which have assessed cell density in some manner.^18^, ^75^ While mean cell densities determined within this work are slightly higher, values reported in literature are within the standard deviation of the current findings.

In addition to differences in cell density between species, an appreciation needs to be afforded to the number of cells to be injected into the disc in terms of how these cell doses compare to the resident cell population and how this translates from a preclinical animal model to a human. From reviewing clinical trial parameters it has been reported that total cell numbers ranging from 1 to 40 million cells are being used (excluding bone marrow concentrates).^5^, ^76^ Furthermore, it appears that just as much variation exists among preclinical studies, with rat models receiving a range of 2–25 thousand cells and goat typically receiving 1–5.5 million cells.^38^, ^39^, ^40^, ^41^, ^57^, ^64^ For the first time, this work sought to contemplate how these ranges compare to the native NP density of the respective species.

It has often been postulated that injecting a significantly large number of cells may exacerbate the biochemical microenvironment, due to competing nutrient demands, and thus the precise cell number being delivered needs to be carefully considered.^19^, ^69^ Despite assuming a homogeneous distribution throughout the NP and maintenance of a discogenic phenotype, prior to this work, the exact number of cells required for functional regeneration was unclear. While delivery of primary NP cells appears safe and has shown some potential for efficacy clinically, there is a clear challenge in obtaining autologous NP tissue and cells for therapeutic use.^77^ Therefore, alternative cell sources such as stem cell therapies have received considerable attention due to their versatility and potential for long term tissue regeneration. Moreover, many studies culture NP cells under anabolic stimulation or coculture (with notochordal/stem cells) to optimize their potential. Many of these cell therapy development studies focus on showing that cells can produce the desired disc‐specific ECM, but it is imperative to identify the timeframe within which these cells can lay down this matrix and subsequently regenerate the IVD. For example, while growth factor stimulation may increase matrix production, it is hypothesized that it may also drive further nutrient deprivation, reducing the overall number of cells which can be injected into the niche. Our recent work showed that priming or preconditioning cells with TGF‐β3 upregulated OCR ~2‐fold in stem cells whereas OCR for differentiated cells had a tendency to decrease.^33^ Therefore, in order to compare alternative cell sources or the effect of anabolic stimulation in silico, differences in the metabolic profile and rates of cells under these conditions need to be fully elucidated.

It was initially hypothesized that animal cells would have higher matrix synthesis rates compared with human cells. However, this was not detected across all ECM components in this study. While collagen production rates for animal cells were significantly higher than human, it was surprising that rat cells had the lowest GAG synthesis rates among the species and no significant difference found between goat and human. Nonetheless, GAG synthesis rates on a per cell basis were relatively low across all species and on average were only ~4% of that of collagen. Not only is this particularly important for the regenerative potential of these cells but compared with collagen and elastin, IVD aggrecan displays a more rapid turnover and is more susceptible to proteolysis due to its less compact structure.^78^ Therefore, overall matrix synthesis and turnover needs to be balanced to have a net regenerative effect. A previous in silico study investigated the effect of increasing GAG synthesis rate and decreasing GAG degradation rate of resident NP cells without further cell injection. In both cases, mildly degenerated discs were predicted to repair effectively but neither could circumvent severely degenerated discs.^53^ Nonetheless, this study was theoretically based and biological treatments to alter synthesis and degradation rates of GAG in vivo need further exploration. The half‐life of GAG turnover used in the current work was derived from experimental literature and had been used in the aforementioned work, together with a previous model by the same authors, where predictions were consistent with experimental measurements.^52^, ^54^, ^55^ However, a caveat of this work is consistent degradation between species, whereas the half‐life of aggrecan is an increasing function of age and may be consistently lower for degenerate human IVD tissue compared with healthy animals.^78^

Nonetheless, the current work showed that based on experimentally determined native GAG synthesis rates, substantial regeneration of the GAG matrix is feasible in rat within a 12‐week timeframe despite injected cell numbers being <5% of the native NP population. Regeneration is also predicted to be feasible in goat within a 12‐month timeframe. However, substantially higher cell doses are necessary. A dose of less than half the NP cell population will only slightly regenerate the GAG matrix, whereas a cell dose of over 1.5 times the NP population was predicted to be necessary to restore GAG to levels close to a healthy goat disc. Meanwhile, human results predict further deterioration of a mildly degenerated Grade III disc over 10 years without a curative cell injection, while a dose of 5 million cells is necessary to prevent GAG content diminishing further in the substantially larger human IVD. Nonetheless, a higher treatment dose of 10–40 million cells is predicted to be capable of initiating regeneration, with the substantial timeframe for functional GAG restoration being years and highly dependent on the number of cells injected clinically.

The IVD develops embryologically from the mesenchyme, with the NP formed by highly specialized notochordal cells which produce the primary matrix components. These cells eventually die off into adulthood being replaced by NP cells when the avascular tissue has thickened and fully formed.^79^, ^80^ Injected cell‐based NP or stem cell therapies are not these highly specialized notochordal cells which have developed in the unique disc microenvironment. While cell therapies may be sufficient to synthesize adequate amounts of GAG in the thinner discs of preclinical animal models, there are significant hurdles for these therapies clinically. For instance, repair using NP transplantation in a small rat model indicated that cell implantation had a significantly greater disc height index compared with the puncture control at just 8 weeks and demonstrated increased central matrix composition through Safranin‐O staining.^38^ Similar follow‐up studies have been performed in clinical trials on the human IVD.^81^, ^82^, ^83^, ^84^ Despite positive results such as increased disc fluid content in treated patients reported at 1‐ and 2‐year follow‐up, there is an overall scarcity in the literature on clinical trials and published reports tend to be vague in their shortcomings and no tremendous breakthroughs have been reported.^5^, ^81^, ^85^

It appears that follow‐up assessments are typically from 6 months to 3 years, whereas the results of this work predict a longer timeframe for detectable functional regeneration which may contribute to ineffective clinical outcomes and conclusions. Furthermore, many studies only report on pain and disability (Visual Analogue Scale and Oswestry Disability Index) at follow‐up.^86^, ^87^ Linking to clinical results which are available using functional MRI assessment, the predicted inefficacy is reflected in a study which revealed only a single patient had a mild improvement at 3 years after a dose of 1 million activated NP cells.^77^ Furthermore, the predicted requisite of at least 10 million cells for functional regeneration is reflected in a study which reported elevated water content at 12 months after a dose of 10 million bone marrow derived mesenchymal stem cells, but no recovery in disc height, which according to predictions in this work is not unsurprising at such an early stage.^88^ Similar promise was reported for eight patients after an injection of 10 million juvenile chondrocytes at an early time point of 12 months.^89^ However, the fundamental question remains whether the microenvironment can sustain the cell doses being delivered. For example, a study reporting cell doses ranging from 15 to 52 million revealed mixed results in a long term feasibility study (4‐ to 6‐year follow‐up).^90^ While a positive correlation between overall improvement and total number of cells injected was reported, the ~52 million cells were administered to a 24‐year‐old patient who exhibited maintenance of disc height while ~28 million cells were administered to a 53‐year‐old patient who exhibited mild progression of IVD degeneration. Therefore, it has been postulated that the inherent microenvironment and thus the optimal cell dose may vary significantly on a patient‐to‐patient basis as reported for in vivo disc measurements.^91^, ^92^

This work does not imply that cell‐based therapies are inevitably “doomed” clinically and that they are not worthy of further pursuit or exploration. More exactly, this work suggests a need to manage expectations in terms of the timeframes needed to achieve successful clinical outcomes while balancing the maximum number of cells, which can be injected without detrimentally perturbing the nutrient microenvironment. Within preclinical models, it was predicted that inducing injury causes nutrient concentrations to increase (due to disrupted cell density), which has been experimentally measured for glucose and lactate in goat models.^93^ As a result, when cells are then injected, the microenvironmental niche is not exacerbated to the same extent as the larger degenerating human IVD. It is speculated that this may explain DiscGenics Inc. moving from 10 million cells in their preclinical rabbit model, to a low dose of 3 million and a high dose of 6 million cells in their ongoing clinical trials (NCT03347708).^94^ While this work predicts that a clinical dose of at least 40 million cells is necessary to achieve healthy Grade II levels of regeneration within 5 years, it also predicts that 40 million cells will adversely affect the nutrient microenvironment with glucose reducing below 1 mM, pH below 6.9 and oxygen below 1% O_2_. As a result, models in this work predict 10 million cells to be an upper limit for cell‐based regeneration. In particular, this is due to lactate accumulation, as the detrimental effect of acidity on cells has been well established in the literature, reporting increased cell death, decreased proliferation, and inhibited anabolic gene expression resulting in decreased matrix accumulation.^33^, ^95^, ^96^ These predictions correlate with our earlier in silico models,^48^ and as mentioned previously, may reflect the clinical shift from early trials using 20–60 million cells per disc to Mesoblast Ltd. progressing with a lower dose of 6 million cells in Phase 3 trials (NCT02412735) following a comparison of low and high doses in Phase 2 trials (NCT01290367).^48^, ^81^, ^88^, ^89^, ^97^

Mechanical loading will vary between different species due to differences in anatomy, size, and loading patterns.^6^, ^11^, ^98^ While the human IVD is subjected to axial compression, due to our bipedal stance, the vast majority of animal models are quadrupedal. Despite this it has been hypothesized that loading exerted in the lumbar region of large animals, such as goat, may be even greater due to the increased complexity of stabilizing a horizontally aligned spine.^6^, ^11^ Furthermore, it has been speculated that intradiscal pressure in small quadrupeds, such as rat, is comparable to that of human since the diameter on which this force is acting is much smaller.^11^ Nonetheless, a recent review reported that only four rat studies actually considered biomechanical factors when assessing tissue regenerative approaches.^42^ Similarly, goat models typically only perform radiography, MRI, and histological analyses to capture degeneration and/or regeneration and generally biomechanical assessment is limited to ex vivo or organ culture models where confounding factors can be limited.^59^ Assessment on how cell based or biological therapies slow degeneration and restore mechanical function after injury are likely to require different experimental setups. Ongoing work is needed to establish long‐term biomechanical stability as well as quantifying changes in animal activity and gait as a consequence of inducing an injury and again following treatment.

While dynamic mechanical loading of the human IVD throughout daily activities is complex and multifactorial, the average modern human lives a relatively sedentary life style with 7–8 h spent lying prone (0.1 MPa) and a further ~8 h in a seated position (0.3–0.8 MPa).^99^ With muscle activity shown to increase intradiscal pressure, it has been cautiously concluded that constantly changing position is important to promote fluid flow.^99^ However, early in vivo studies found no significant effect of dynamic loading or “pumping” due to exercise on nutrient transport of small glucose and oxygen molecules in canine discs or rabbit spines which had undergone flexion and extension.^100^, ^101^ Despite this, another rabbit study demonstrated that diffusion through the CEP can be modestly enhanced by forced convection under low‐rate dynamic loading.^102^ Moreover, a study by Salvatierra et al.^31^ investigated the effect of dynamic compression on disc cell metabolism. The authors found that while compressive loading significantly increased GCR (61%) and LPR (52%) in AF cells, the effect on NP cells was not statistically significant. Overall the metabolic rates measured were higher than in the current work. This may be due to differences in the experimental setup such as cell‐laden agarose constructs versus cellular aggregates, supraphysiologically high nutrient conditions or notochordal cells isolated from relatively young pigs (4–6 months) which have been reported to be both more metabolically active and sensitive to nutrient levels compared with non‐notochordal species.^25^

Comparison of metabolic rates between species in this work found that human cells (both NP and AF) had significantly lower OCRs than animal disc cells, signifying lower respiration in the human cells. This supports previous evidence that disc cells acquire most of their energy from glycolysis due to the large human IVD being a challenged oxygen environment.^17^, ^20^ Meanwhile, animal AF cells having a higher OCR than their corresponding NP cells may be a result of an oxygen gradient through the tissue in vivo, with more aerobic respiration occurring under higher oxygen levels. A positive Pasteur effect describes the phenomenon where glycolysis (in this case LPR measurement) is suppressed by high oxygen concentrations and subsequently results in an increase in respiration. While this was detected for rat with NP cells having higher LPR than AF cells, goat NP cells did not appear to have higher rates of LPR than AF cells despite their lower OCR. However, this must be approached with some caution as this study did not directly investigate the effect of nutrient concentrations on metabolic rates for NP and AF cells. Several studies have observed a positive Pasteur effect in NP and AF cells (canine, porcine, and bovine),^17^, ^20^, ^25^, ^27^ while a recent study with porcine cells observed a positive Pasteur effect in AF cells but not NP cells.^28^ Additionally, below 8% O_2_, oxygen has been shown to decrease the rate of LPR, while above 10% O_2_ the effect of oxygen was insignificant (i.e., a negative Pasteur effect).^22^ It is speculated that the large standard deviation in AF rates measured may be due to not separating the AF into inner and outer regions. Furthermore, it has been demonstrated that human NP and AF cells have different GLUT expression profiles suggesting regional differences in the metabolic nature of the human IVD.^65^ However, this study was unable to detect a significant difference between human NP and AF in particular. Together, this may be due to challenging identification of distinct tissue regions in samples from microdiscectomy surgeries. The lack of specific markers for AF cells has hampered protocols to confirm distinct NP and AF cell populations. However, new markers such as CD146 and Mohawk (MKX) have been identified to characterize AF phenotype and provide opportunities to identify more distinct cell populations.^103^

When compared with the literature for human disc cells, OCR measured in this study is significantly lower. Cisewski et al.^26^ culture expanded healthy and degenerated human disc cells in monolayer to achieve a sufficient number of cells to carry out investigations into OCR on a cell suspension. However, culture expansion has been shown to shift chondrocytes from a glycolytic to an oxidative energy metabolism within 7 days in vitro.^32^ The current study is the first to measure metabolic rates of disc cells in a 3D spheroid configuration where cell‐to‐cell interaction and pericellular matrix deposition enhance cell attachment, proliferation, matrix production, and phenotype expression.^104^ Additionally, this microspheroid culture system was favored as it can be used with low seeding densities, which minimizes nutrient gradients and reduces the need for culture expansion, thus limiting alterations to the cell metabolism and loss of a disc cell phenotype.

A limitation of the rates in this work is that viability was not quantitatively assessed to adjust the normalization by total DNA content and may result in rates being calculated to a lower level. Nonetheless, using the Seahorse analyzer results in a high throughput and significantly more sensitive system, with a 2.3 μL microchamber created around each individual spheroid as opposed to the 175 μL to 4 mL metabolic chambers used previously.^22^, ^24^, ^26^ In order to calculate GCR of disc cells for in silico modeling, an assumption of 2:1 glycolysis was made. Although there is literature to suggest this ratio can change, it does not alter significantly enough to invalidate this assumption. Overall, the higher glycolytic rates for disc cells, measured in terms of LPR in this work, indicate a less efficient pathway of energy production. Despite similar rates of GAG production between species, higher OCR in animal cells indicate more efficient energy production, with aerobic respiration producing 36 ATP molecules per molecule of substrate compared with 2 ATP through anaerobic glycolysis.

Taken together, this work provides insight into the cell number capable of surviving and initiating repair without exacerbating the microenvironmental niche. It has previously been implied that the impoverished nutrient levels impose an upper limit on the cell number which can be implanted.^48^, ^68^, ^70^, ^84^, ^88^ However, this is the first work to theoretically propose a cell dose which attempts to balance both the biochemical microenvironment and sufficient matrix synthesis to initiate functional repair. Furthermore, this study informs the timeline within which positive changes could be expected and detected clinically. The main findings of this work were that these in silico models compare favorably to the preclinical literature in terms of the capabilities of animal regeneration. However, they predict very long timeframes (of the order of years) for regeneration in the large human IVD. It is speculated that this may explain the variable results emerging from trials and the failure of cell‐based regeneration to be adopted clinically.^5^, ^76^, ^105^, ^106^, ^107^, ^108^, ^109^

Despite the important insights provided in this work, it is important to bear in mind that cell‐based therapies first and foremost initiate matrix deposition and compositional changes to the disc and this is the extent of predictions within this work. This work is not capable of predicting complete restoration of the IVD structure and/or biomechanical function. However, IVD degeneration and its associated spinal pain is a complex multifactorial process and in order to be clinically viable, therapeutic strategies need to alleviate pain perception. As mentioned previously, more thorough assessments on how cell‐based therapies slow degeneration and restore mechanical function after injury are required. Additionally, there is currently no direct measurement of pain, only of the perceived disability it causes clinically, while preclinical animal models are also unable to accurately capture the level of ongoing pain which they are suffering.^11^ Nonetheless, in order to prevent occurrences which lead to pain, regeneration of the GAG matrix and preservation of a healthy functional IVD is important.^110^ Ongoing work is needed to accurately evaluate and establish long‐term biomechanical stability of biological treatments in both animal models and clinically.

While this work enables the research field and clinicians to manage expectations on cell‐based regeneration, it suggests our credulousness that cells alone will be sufficient to establish de novo GAG matrix and bring about “functional change” to a degenerating disc. Perhaps cell therapies are better placed as a preventative rather than curative therapy, where a successful outcome is simply impeding further degeneration in the long term. As a result of elucidating the temporal and scale limitations of relying solely on cells, this work advocates the prospects of alternative therapeutic strategies such as reprogramming cells through microRNA and gene engineering approaches or preconditioning and priming strategies.^33^, ^70^, ^111^ Moreover, combining cell approaches with biomimetic biomaterials may be a more potent therapy to restore or recreate the structural and biochemical composition of the damaged tissue within an expedited timeframe.^112^

## 5 CONCLUSION

For the first time, this work corroborates what has long been postulated, that the scale effect of cell‐based human IVD regeneration is not trivial. The IVD is the largest avascular structure in the body with significant diffusional distances which hamper the nutrient supply to both inherent disc cells and injected therapeutic cells. This work presents in silico models which correlate favorably to preclinical literature in terms of the capabilities of animal regeneration and predicts that compromised nutrition is not a significant challenge in small animal discs. On the contrary, this study highlights a very fine clinical balance between an adequate cell dose for sufficient repair, through de novo matrix deposition, without exacerbating the human microenvironmental niche. While these findings help to explain the failed translation of promising preclinical data and the stunted results emerging from clinical trials at present, they also enable the research field and clinicians to manage expectations on cell‐based regeneration. Furthermore, these results may inform the design of clinical trials in terms of more long‐term follow‐up assessment (over a number of years) for positive functional change. Additionally, as computing power and software capabilities increase in the future, it is conceivable that generation of patient‐specific models could be used for patient assessment, as well as pre‐ and intraoperative planning.

## AUTHOR CONTRIBUTIONS

Emily E. McDonnell and Conor T. Buckley contributed substantially to the conception and design of the work. Emily E. McDonnell performed the acquisition and interpretation of literature data, computational modeling, analysis presentation and interpretation of results, drafting of the article, revising it critically, and final approval. Niamh Wilson contributed to acquisition of laboratory data. Marcos N. Barcellona and Tara Ní Néill contributed to the acquisition of laboratory samples. Jessica Bagnall contributed to the MRI imaging processing. Pieter A. J. Brama contributed to the acquisition of animal samples. Gráinne M. Cunniffe, Stacey L. Darwish, and Joseph S. Butler contributed to the acquisition of surgical samples from the Mater Misericordiae University Hospital. Conor T. Buckley, as the overall project funding holder, takes responsibility for the integrity of the work from inception to finalized article, provided substantial contribution to data interpretation and presentation. Emily E. McDonnell and Conor T. Buckley drafted the article. All authors clinically revised the article and approved the final version.

## CONFLICT OF INTEREST STATEMENT

Conor T. Buckley is an Editorial Board member of *JOR Spine* and co‐author of this article. They were excluded from editorial decision‐making related to the acceptance of this article for publication in the journal.

## Supporting information


**FIGURE S1.** Preferred Reporting Items for Systematic Reviews and Meta‐Analysis (PRISMA) diagram indicating screening process and exclusion criteria. Eighty articles were reviewed for rat tail models and 15 articles for goat lumbar models.
**FIGURE S2.** Geometrical analysis of goat lumbar and rat caudal disc using macroscopic and microscopic image analysis, respectively. (A) Goat lumbar discs L1‐2 to L5‐6 were dissected in the transverse and sagittal plane to determine the anterior to posterior distance, lateral width, and disc heights across the midsection. (B) Histologically stained (H&E: hematoxylin and eosin; PSR&AB: picrosirius red and alcian blue) transverse sections of goat lumbar discs to confirm the interface of the nucleus pulposus (NP) and annulus fibrosus (AF) through the change in matrix composition. (C) Rat caudal discs Cd3‐4 to Cd9‐10 were microsectioned in the sagittal plane and histologically stained to evaluate the full disc diameter, NP diameter and the central disc height.
**FIGURE S3.** Agarose microwell array fabrication and the formation of disc spheroids. (A) Geometry of a 3D printed stamp and the process to be used to create 69 microwells array in molten 2% agarose in each well of a 24‐well plate. The cross‐section sketch highlights the dimensions of each individual microwell. (B) Microscope image of the negative mold left in the solidified agarose after the removal of the stamp. (C) Schematic of the steps involved in creating the disc spheroids within the agarose microwell array. Components created with BioRender.com.
**FIGURE S4.** In silico modeling of the microwell culture system to inform the external boundary concentrations necessary to create a physiologically relevant microenvironment within the spheroids. Predicted average glucose and pH concentrations within spheroids, which underwent a culture media exchange every 3 days or a daily media exchange. (B) Contour plots of glucose, pH and oxygen distributions within three microwells of the agarose array for rat, goat, and human (top to bottom). (C) Average concentrations predicted within the spheroids presented above, with the standard deviation accounting for the minor variation in diameter of the three spheroids presented for each species.
**FIGURE S5.** An example of raw measurements from the Seahorse XFe96 analyzer showing simultaneous measurement in real time of the reduction in oxygen and pH level for rat, goat, and human nucleus pulposus cells. Levels reduce over a 30 min. measurement period, before the sensor cartridge rises to allow oxygen to reinfiltrate and fresh media exchange (causing the pH to rise). The graphs show four repeated measurement periods for more than 25 spheroids per prep/donor prior to normalization for cell number.
**FIGURE S6.** Towards experimental validation of *in* silico modeling of the nutrient microenvironment in preclinical animal models. (A) Preliminary experimental measurement of oxygen and pH values in the center of rat caudal discs (*N* = 3) using probing technology and compared with predicted in silico results. (B) Preliminary experimental measurement of glucose and pH values in the center of goat lumbar discs (*N* = 3) using biochemical and probing technology, respectively. Measured values (black) are compared with predicted in silico results for each metabolite (colored).
**TABLE S1.** Boundary concentrations used in silico at the nucleus pulposus (NP)/cartilage endplate (CEP) interface and the periannular surface of the annulus fibrosus (AF) for rat, goat, and human.
