# Supplementary material for: Preclinical to clinical translation for intervertebral disc repair: Effects of species‐specific scale, metabolism, and matrix synthesis rates on cell‐based regeneration
Source: JOR Spine. 2023 Sep 7;6(3):e1279. doi: 10.1002/jsp2.1279 (PMC10540833; doi:10.1002/jsp2.1279)
Supplement: Supplementary file 1 — FIGURE S1. Preferred Reporting Items for Systematic Reviews and Meta‐Analysis (PRISMA) diagram indicating screening process and exclusion criteria. Eighty articles were reviewed for rat tail models and 15 articles for goat lumbar models. FIGURE S2. Geometrical analysis of goat lumbar and rat caudal disc using macroscopic and microscopic image analysis, respectively. (A) Goat lumbar discs L1‐2 to L5‐6 were dissected in the transverse and sagittal plane to determine the anterior to posterior distance, lateral width, and disc heights across the midsection. (B) Histologically stained (H&E: hematoxylin and eosin; PSR&AB: picrosirius red and alcian blue) transverse sections of goat lumbar discs to confirm the interface of the nucleus pulposus (NP) and annulus fibrosus (AF) through the change in matrix composition. (C) Rat caudal discs Cd3‐4 to Cd9‐10 were microsectioned in the sagittal plane and histologically stained to evaluate the full disc diameter, NP diameter and the central disc height. FIGURE S3. Agarose microwell array fabrication and the formation of disc spheroids. (A) Geometry of a 3D printed stamp and the process to be used to create 69 microwells array in molten 2% agarose in each well of a 24‐well plate. The cross‐section sketch highlights the dimensions of each individual microwell. (B) Microscope image of the negative mold left in the solidified agarose after the removal of the stamp. (C) Schematic of the steps involved in creating the disc spheroids within the agarose microwell array. Components created with BioRender.com. FIGURE S4. In silico modeling of the microwell culture system to inform the external boundary concentrations necessary to create a physiologically relevant microenvironment within the spheroids. Predicted average glucose and pH concentrations within spheroids, which underwent a culture media exchange every 3 days or a daily media exchange. (B) Contour plots of glucose, pH and oxygen distributions within three microwells of the agarose [file JSP2-6-e1279-s001.docx]

Supplementary datasets associated with this article can be found in an online repository at DOI:10.5281/zenodo.7670645.


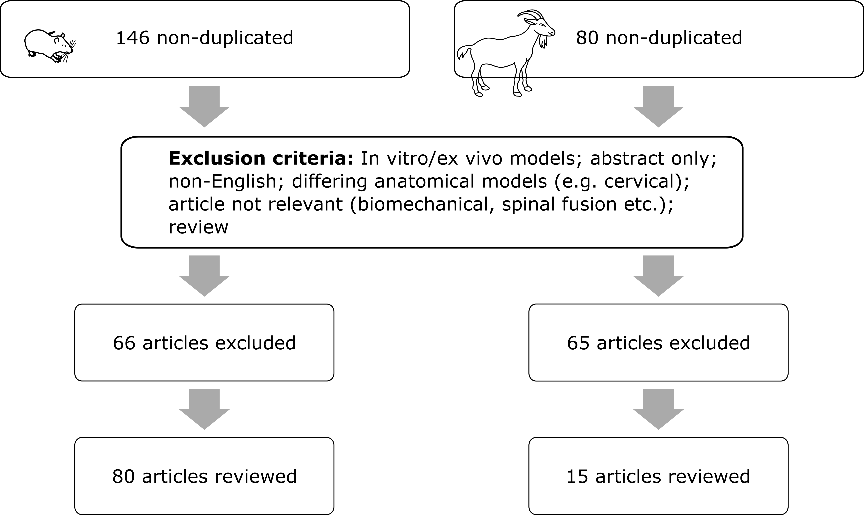


Figure S1. Preferred Reporting Items for Systematic Reviews and Meta-Analysis (PRISMA) diagram indicating screening process and exclusion criteria.

80 articles were reviewed for rat tail models and 15 articles for goat lumbar models.


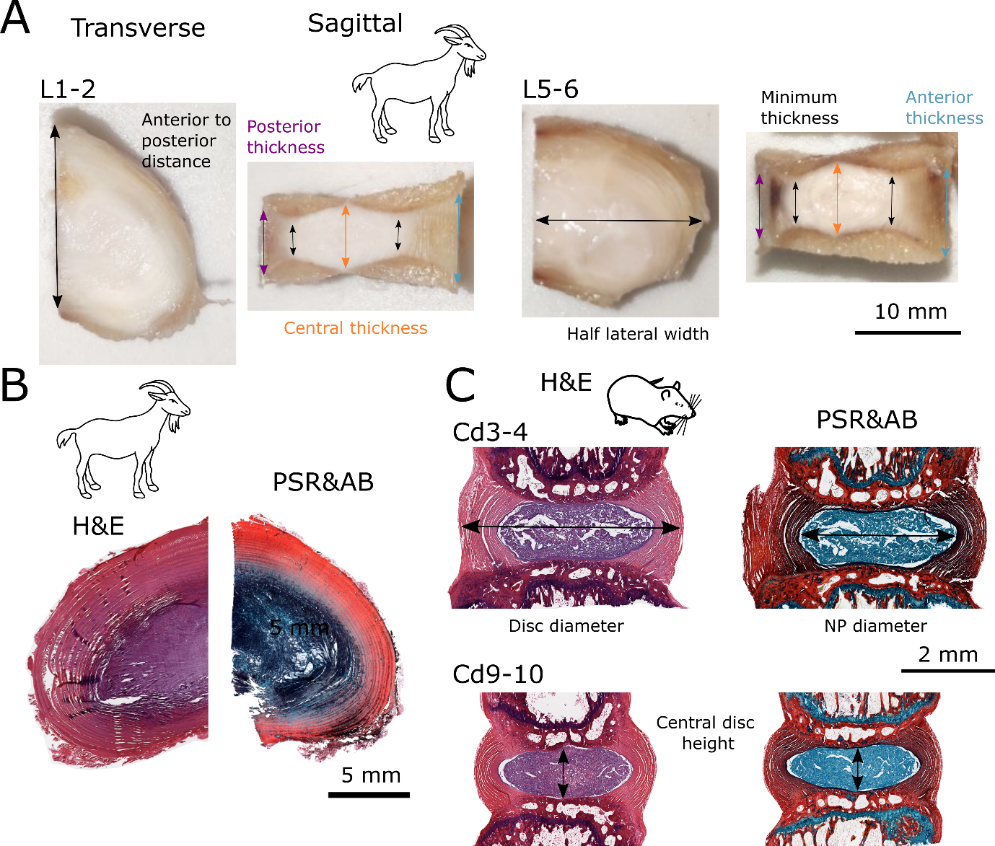


**Figure S2.** Geometrical analysis of goat lumbar and rat caudal disc using macroscopic and microscopic image analysis, respectively.

**(A)** Goat lumbar discs L1-2 to L5-6 were dissected in the transverse and sagittal plane to determine the anterior to posterior distance, lateral width, and disc heights across the midsection. **(B)** Histologically stained (H&E: hematoxylin and eosin; PSR&AB: picrosirius red and alcian blue) transverse sections of goat lumbar discs to confirm the interface of the nucleus pulposus (NP) and annulus fibrosus (AF) through the change in matrix composition. **(C)** Rat caudal discs Cd3-4 to Cd9-10 were micro-sectioned in the sagittal plane and histologically stained to evaluate the full disc diameter, NP diameter and the central disc height.


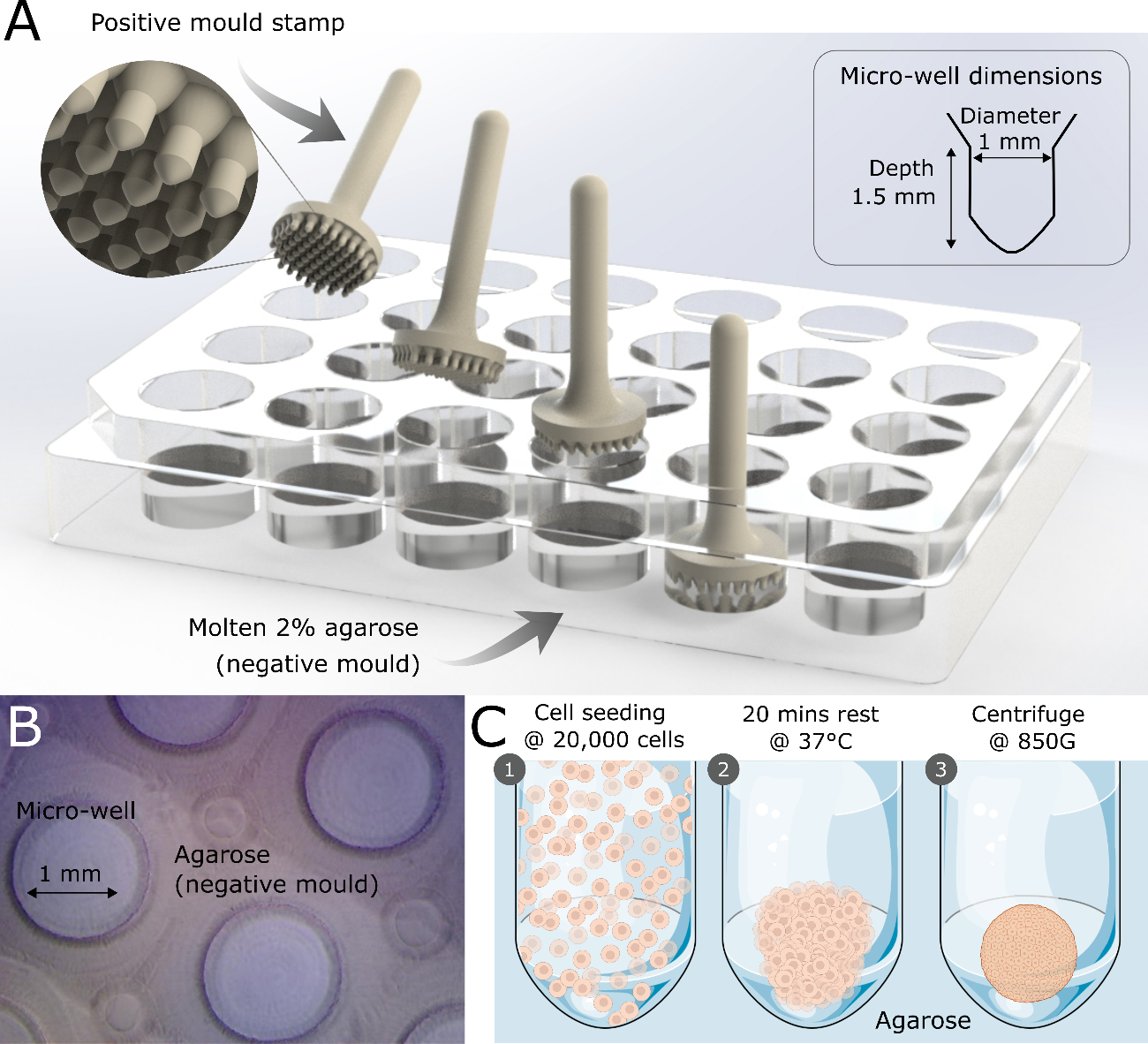


Figure S3. Agarose microwell array fabrication and the formation of disc spheroids.

(A) Geometry of a 3D printed stamp and the process to be used to create 69 micro-wells array in molten 2% agarose in each well of a 24-well plate. The cross-section sketch highlights the dimensions of each individual microwell. (B) Microscope image of the negative mould left in the solidified agarose after the removal of the stamp. (C) Schematic of the steps involved in creating the disc spheroids within the agarose microwell array. Created with [BioRender.com](https://biorender.com/).


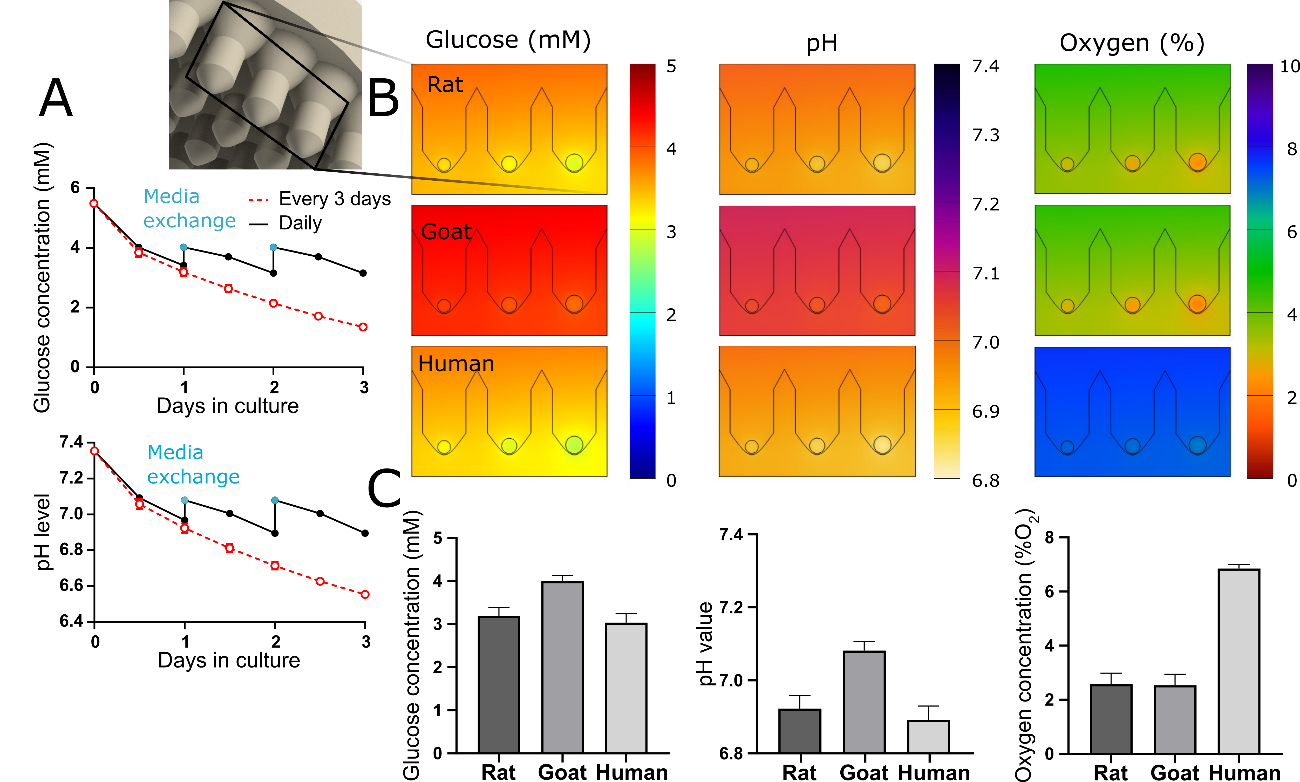


Figure S4. *In-silico* modelling of the microwell culture system to inform the external boundary concentrations necessary to create a physiologically relevant microenvironment within the spheroids.

Predicted average glucose and pH concentrations within spheroids which underwent a culture media exchange every three days or a daily media exchange. (B) Contour plots of glucose, pH and oxygen distributions within three microwells of the agarose array for rat, goat and human (top to bottom). (C) Average concentrations predicted within the spheroids presented above, with the standard deviation accounting for the minor variation in diameter of the three spheroids presented for each species.


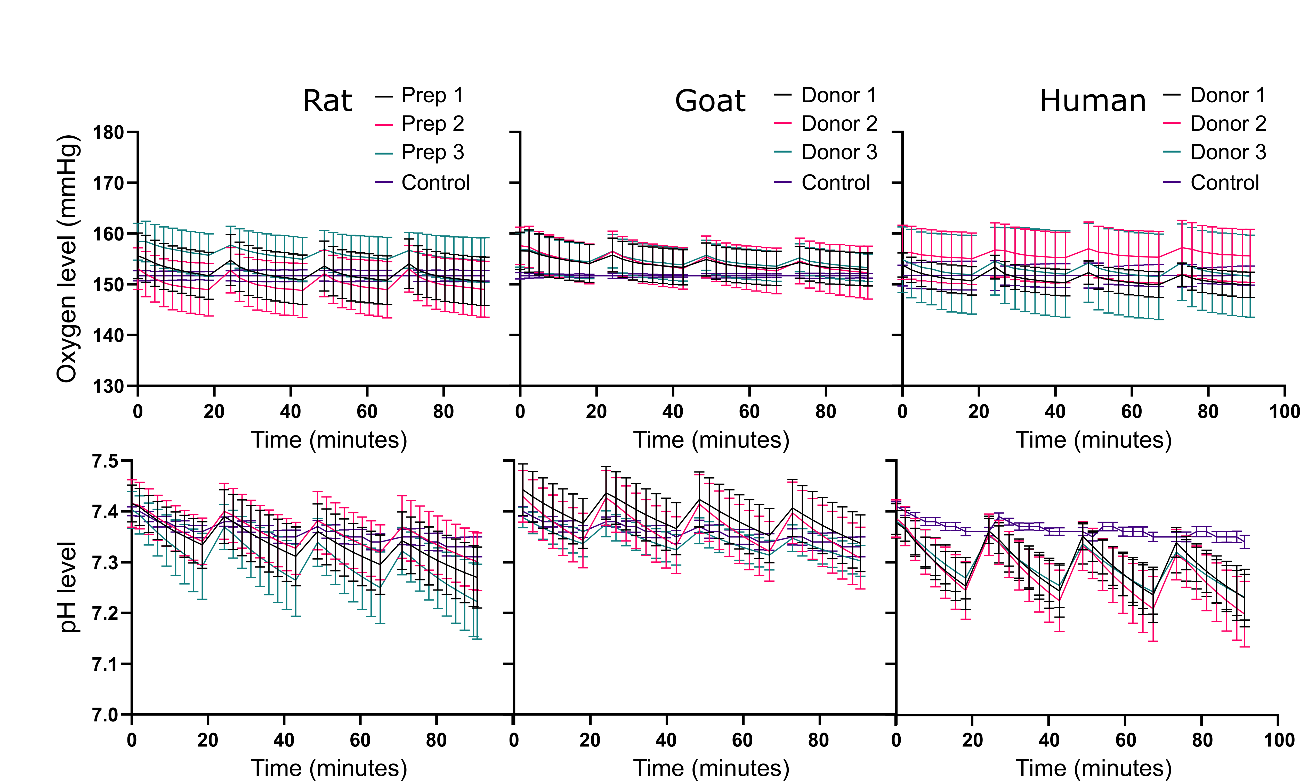


Figure S5. An example of raw measurements from the Seahorse XFe96 analyser showing simultaneous measurement in real time of the reduction in oxygen and pH level for rat, goat, and human nucleus pulposus cells.

Levels reduce over a 30 min. measurement period, before the sensor cartridge rises to allow oxygen to re-infiltrate and fresh media exchange (causing the pH to rise). The graphs show four repeated measurement periods for more than 25 spheroids per prep/donor prior to normalisation for cell number.


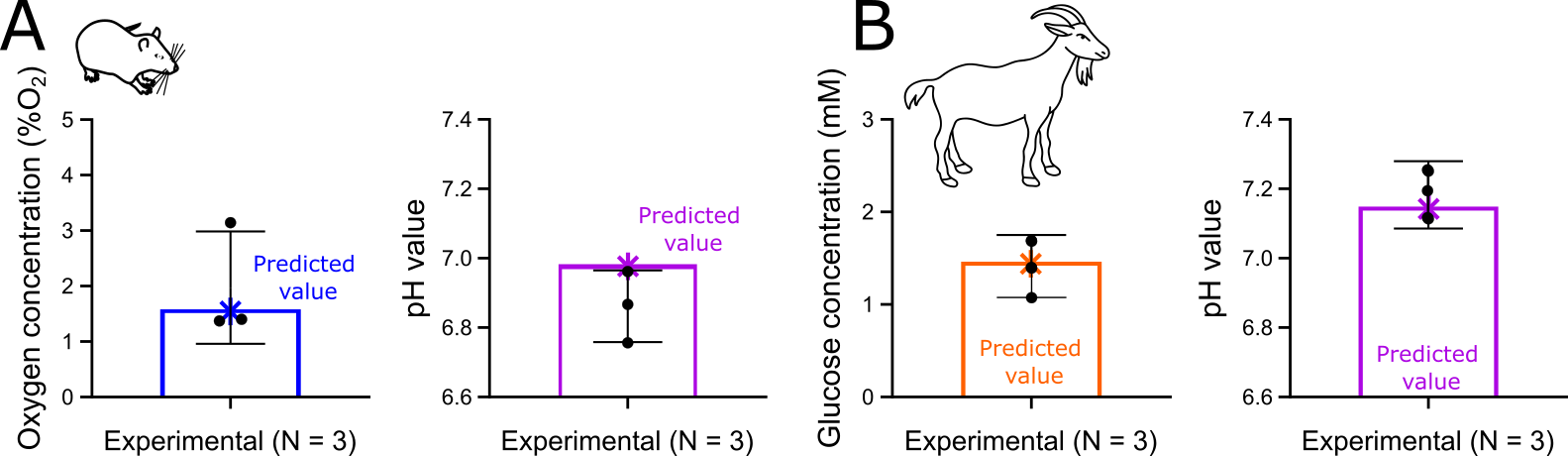


Figure S6. Towards experimental validation of in-silico modelling of the nutrient microenvironment in pre-clinical animal models.

(A) Preliminary experimental measurement of oxygen and pH values in the centre of rat caudal discs (N = 3) using probing technology and compared to predicted *in-silico* results. (B) Preliminary experimental measurement of glucose and pH values in the centre of goat lumbar discs (N = 3) using biochemical and probing technology, respectively. Measured values (black) are compared to predicted *in-silico* results for each metabolite (coloured).

Table S1. Boundary concentrations used *in-silic*o at the nucleus pulposus (NP)/cartilage endplate (CEP) interface and the periannular surface of the annulus fibrosus (AF) for rat, goat, and human.

|  | Rat | | Goat | | Human | |
| --- | --- | --- | --- | --- | --- | --- |
|  | NP | AF | NP | AF | NP | AF |
| *Gluc (mM)* | 3.50^a^ | 3.95^1^ | 2.90^a^ | 3.90^2^ | 3.04^a^ | 4.75^3^ |
| *O_2_ (%O_2_)^b^* | 2.22 | 5.92 | 1.85 | 5.92 | 1.48 | 5.92 |
| *Lac (mM)* | 3.30^d^ | 1.98^c^ | 3.30^d^ | 1.98^c^ | 3.30^d^ | 1.98^c^ |

^a^ Concentrations at the NP-EP interface were estimated based on the reduction in glucose through the EP modelled in our previous work.^4^

^b^ Periannular oxygen concentration was approximated from the literature and iteratively adjusted according to preliminary experimental validation of the models. Concentrations at the NP-EP interface were estimated based on reduction through the EP and iterative experimental validation.

^c^ Periannular lactate concentration assumed to be that of typical blood plasma (pH 7.35 – 7.45).

^d^ Concentrations at the NP-EP interface were estimated based on the reduction in lactate through the EP modelled in our previous work together with iterative experimental validation. ^4^
